# Chemical characterization and encapsulation of *Ganoderma pfeifferi* extract with cytotoxic properties

**DOI:** 10.3389/fphar.2025.1526502

**Published:** 2025-01-23

**Authors:** Jan Šťastný, Ángela Morellá-Aucejo, Tomáš Skala, Andrea Bernardos, Petr Maršík, Araceli Lérida-Viso, Jaroslav Matějka, Anna Mascellani Bergo, María Dolores Marcos, Ramón Martínez-Máñez, Ivan Jablonský, Pavel Klouček

**Affiliations:** ^1^ Department of Food Science, Faculty of Agrobiology, Food and Natural Resources, Czech University of Life Sciences Prague, Prague, Czechia; ^2^ Instituto Interuniversitario de Investigación de Reconocimiento Molecular y Desarrollo Tec-nológico (IDM), Universitat Politècnica de València-Universitat de València, Valencia, Spain; ^3^ Unidad Mixta UPV-CIPF de Investigación en Mecanismos de Enfermedades y Nanomedicina, Universitat Politècnica de València-Centro de Investigación Príncipe Felipe, Valencia, Spain; ^4^ CIBER de Bioingeniería, Biomateriales y Nanomedicina (CIBER-BBN), Instituto de Salud Carlos III, Valencia, Spain; ^5^ Department of Horticulture, Faculty of Agrobiology, Food and Natural Resources, Czech University of Life Sciences Prague, Prague, Czechia

**Keywords:** HeLa, bioactivity, reishi mushroom, antiproliferative activity, applanoxidic acids

## Abstract

Mushrooms of the genus *Ganoderma* are known for diverse biological activities, demonstrated both traditionally and experimentally. Their secondary metabolites have shown cytotoxic potential across different cancer cell lines. Besides exploration of the most active components in different species or genotypes, new formulation techniques are in development. In recent years, there has been a growing interest in the use of nanomaterials because of significant potential for pharmacology applications as substance carriers. Applying nanoparticles may enhance the medicinal effect of the mushroom substances. This study investigated the cytotoxic properties of *Ganoderma* species methanolic extracts against the HeLa cancer cell line. Notably, the extract obtained from *Ganoderma pfeifferi* demonstrated the highest activity and was further used for encapsulation within synthesized mesoporous silica nanoparticles MCM-41. Subsequently, the cytotoxic effect of the loaded MCM-41 to the free form of extract was compared. The obtained results indicate successful encapsulation, and similar activity comparing encapsulated form to free extracts (IC_50_ 16.6 μg/mL and 20.5 μg/mL, respectively). In addition, the four unique compounds were identified as applanoxidic acid A, applanoxidic acid G, ganoderone A, and ganoderone B in the *G. pfeifferi.* This study is an essential prerequisite for further steps like nanoparticle functionalization for sustained or on-command delivery of these natural extracts.

## 1 Introduction

Throughout history, mushrooms have played an active role in human culture. They are highly valued as a tasty source of minerals, dietary fiber, vitamins, and proteins, with a relative high abundance of essential amino acids (Cheung, 2010). Beyond their nutritional value (Wasser, 2011) and ritual use (Varghese et al., 2019), certain mushrooms are known for their health benefits, leading to their use in treating various diseases (Wasser, 2011). *Pleurotus* mushrooms, for instance, have been part of traditional folk medicine (Rama et al., 2012). Traditional Chinese medicine documents the use of mushrooms such as Shiitake (*Lentinula edodes*) or Reishi/Lingzi mushrooms (*Ganoderma lucidum*), which are believed to treat a wide spectrum of diseases (Money, 2016). Reishi mushrooms (*Ganoderma* spp.) are used in the form of powder extract, tea, or dietary supplements (Ahmad et al., 2021) and have a long history of use in East Asian herbal medicine (Shiao, 2003). The health-promoting effects of mushrooms are often linked to bioactive compounds they produce as secondary metabolites (Money, 2016). The mushroom genera *Fomes*, *Agaricus*, *Flammulina*, *Antrodia*, *Trametes*, *Lactarius*, *Pleurotus,* and *Ganoderma* have been investigated by multiple studies regarding their medicinal qualities (Patel and Goyal, 2012). *Ganoderma* spp. are known for their rich secondary metabolism covering over 400 bioactive compounds, with triterpenoids and polysaccharides being the most significant in relation to pharmacological activity (Gündoğdu and Özenver, 2023). These compounds have shown cytotoxic potential *in vitro* across different cancer cell lines such as the pancreas, lung, colon, skin, breast, prostate, and liver (Sharma et al., 2019). Triterpenoids have been associated with anti-tumor benefits (Gündoğdu and Özenver, 2023), cytotoxic qualities (Kuo et al., 2016) and the ability to induce apoptosis (Gündoğdu and Özenver, 2023). Approximately 150 biologically active triterpenoids, such as ganoderic acids, lucidenic acids, ganoderiols, ganodermatriol, lucialdehydes, lucidumols (Gündoğdu and Özenver, 2023), and ganoderans have been identified in extracts of *Ganoderma lucidum* and some of them have been associated with anti-tumor benefits (Cör, Knez, and Knez Hrnčič, 2018). *G. pfeifferi*, another species of the *Ganoderma* genus, has been identified to contain a whole range of bioactive compounds, including triterpenes such as ganoderone A, lucialdehyde B (Elkhateeb, 2023), ganodermadiol, lucidadiol, applanoxidic acid G (Mothana et al., 2003), and ganomycins A and B (Niedermeyer et al., 2013). This highlights mushrooms as potential sources of novel metabolites for drug development (Money, 2016). Some genera of mushrooms with potential medicinal effects may be used as a new drug class, so-called mushroom pharmaceuticals (Chang and Wasser, 2018).

Cancer, following cardiovascular diseases, stands as the second most prevalent cause of mortality in the global population (Roth et al., 2018). In 2020, the worldwide estimated number of cancer cases was between 19.0 and 19.6 million, with the associated mortality ranging from 9.7 to 10.2 million individuals (Ferlay et al., 2021). Commonly administrated drugs for cancer treatment lack specificity (Patel and Goyal, 2012) and conventional methods to fight cancer, such as radiotherapy and chemotherapy, are associated with numerous side effects and health complications for patients. There is a growing interest in exploring new, less harmful alternative treatments and therapies (Sarma, Datta, and Saha, 2018). One of the most promising lines of research in the field of cancer treatment is the application of nanotechnology (Estepa-Fernández et al., 2021), which employs different types of nanomaterials capable of serving as carriers. This innovative strategy avoids adverse effects of the drugs and drug deactivation, and reduces the dosage needed for treatment through precise targeting and improved drug solubility (Pastorin, 2009). This concept is well-suited for the food industry, especially in the food supplement and nutraceutical fields (Peters et al., 2016). Many applications have been tested, including enhanced cytotoxic properties of well-known plant bioactive compounds like curcumin and resveratrol (Paolino et al., 2021).

Among the various nanodevices, mesoporous silica nanoparticles (MSNs) have been used in recent years as potential drug carriers that provide unique features such as stability, biocompatibility, large load capacity (Muñoz et al., 2003; Schmaljohann, 2006; Vallet-Regi et al., 2001; Vallet-Regi, Balas, and Arcos, 2007), cargo protection and preservation, and the ability to attach the molecular gate preventing cargo release until a triggering stimulus is applied (Kailasapathy, 2009; Prado, Moura, and Nunes, 2011; Sun-Young et al., 2012). Recently, controlled release through gated materials has been an emerging and groundbreaking concept implemented in drug delivery (Estepa-Fernández et al., 2021; Lérida-Viso et al., 2022). MSNs can be produced in different sizes with pores ranging from 2 to 10 nm, each tailored for specific applications (Kailasapathy, 2009; Prado et al., 2011; Sun-Young et al., 2012).

In this scenario, the objective of the study was to select the most interesting strains among different species of the genus *Ganoderma* based on untargeted LC-MS analysis and cytotoxicity against HeLa cells. For the most effective strain, we aimed to prepare an encapsulated form of its methanolic extract in MSNs, compare its effectiveness against HeLa and healthy fibroblasts, and provide chemical characterization of the main components.

## 2 Materials and methods

The experimental workflow is pictured in the flowchart in Supplementary Figure S1.

### 2.1 Samples and their cultivation

The objects of the study were chosen from the collection at the Crop Research Institute in Prague. It included *Ganoderma lucidum* var. KZ74 **(KZ74)** *, Ganoderma lucidum* var. KZ76 **(KZ76)** and *Ganoderma pfeifferi* **(GPFE)** *, Ganoderma applanatum* var. 2023 **(2023),** *Ganoderma lucidum* var. 338 **(G338),** *Ganoderma applanatum* **(GAPP),** *Ganoderma. lucidum* var. GA2 **(GLA),** *Ganoderma sichuanense* (linghzi) **(GLIN),** *Ganoderma oregonense* **(GORE),** *Ganoderma resinaceum* **(GRES),** *Ganoderma tsugae* **(GTSU)**. The samples were cultivated and harvested by the Department of Horticulture at the Czech University of Life Sciences Prague (CZU). The cultivation process of samples was as follows. 2,500 g of pasteurized mixture (90°C for 24 h) of broad-leaved saw dust enriched with 20% wheat middlings and water (65%–68% relative humidity) were filled into polypropylene bags. Five per cent of grain spawn of *Ganoderma* mushroom was added into a cooled substrate and mixed equally. The mushrooms were cultivated at 25°C–30°C for 21–27 days in relative humidity 80%–85% and collected after ripening.

### 2.2 Preparation of methanolic extract

After harvesting, the mushroom samples were freeze-dried for 3 days, ground into a powder and stored at room temperature in a dark place. 80% methanol was selected for extraction based on preliminary experiments (Supplementary Table S1). In comparison with trichlormethane or hexane it had higher extraction yield. Per 2.0 g of powdered sample, 24 mL of 80% methanol was used (min. 99.9%, Scharlau, Barcelona, Spain) on an orbital laboratory shaker at 210 RPM for 30 min. Subsequently, the extracts were sonicated for 2 min and centrifuged at 1.12·10^4^ × G for 10 min. Supernatants (extracts) were transferred into the evaporation flask, and the solid (residue) was subsequently reextracted with the identical extraction procedure. Both supernatants were mixed and evaporated at 30°C at a low pressure on a rotary evaporator. The flasks with evaporated extracts were weighted, and then the extracts were resuspended into dimethyl sulfoxide (min 99.9%, Scharlau, Barcelona, Spain) to concentration 12.8 mg/mL, which was used as a stock solution for subsequent experiments.

### 2.3 Synthesis and characterization of nanoparticles

The synthesis of MSNs, type MCM-41, was conducted as follows: 1.0 g of *N*-cetyltrimethylammonium bromide (1.37 mmol, for molecular biology ≥99%, Sigma-Aldrich, St. Louis, MO, United States) was added into 480 mL of deionized water. Then, 3.5 mL of 2 M sodium hydroxide (≥99.0%, Labkem, Barcelona, Spain) and a magnetic stirrer were added to the solution. The mixture was mixed and heated to 80 °C, and then 5.0 mL of tetraethyl orthosilicate (12.85 mmol, reagent grade 98%, Sigma-Aldrich, St. Louis, MO, United States) was added dropwise to the mixture. The mixture was then stirred for 2 h at 80 °C and a white precipitate occurred. The formed precipitate was separated using centrifugation at 1.12·10^4^ × G and washed with deionized water until the pH of the supernatant was neutral. The synthesized material was dried in an oven overnight (at 70°C) to obtain “as made” MSNs. Then, the material was calcinated (at 550°C) in the oven within a standard atmosphere for 5 h to eliminate the template phase and obtain the final scaffold (**MSN**_**cal**_).

In order to load the nanoparticles with the extract, a process of loading was executed. Firstly, a portion of the 80% mushroom methanolic extract was dried and redissolved in methanol in order to carry out the impregnation. In a subsequent impregnation procedure, 500 mg of **MSN**_**cal**_ was mixed with the mushroom methanolic extract and putted in an oven at 70°C to evaporate the residual methanol solvent. The nanoparticles were then dried, and the entire extract was loaded onto the dried nanoparticles, resulting in the formation of **MSN**_**GPFE**_.

MSNs were characterized using standard methods, including transmission electron microscopy (TEM), N_2_ adsorption-desorption isotherms, powder X-ray diffraction (PXRD), infrared spectroscopy (FT-IR), ζ (zeta) potential, dynamic light scattering (DLS), and thermogravimetric analysis (TGA). The TEM images were captured using a JEOL JEM-1400 Flash Electron microscope (JEOL Europe SAS, Croissysur-Seine, France) operating at 120 kV. N_2_ adsorption-desorption isotherms were obtained using a Micromeritics TriStar II Plus automated analyzer (Micromeritics Instrument Corporation, Norcross, United States). Prior the measurement, the samples were degassed at 90°C in a vacuum overnight, and measurements were taken at 77 K. PXRD measurements were recorded on a Bruker D8 Advance diffractometer (Bruker Corporation, Billerica, Massachusetts, United States) using CuKα radiation at low angles (1.3 < 2θ < 8.3), with steps of 0.04° and 3 s per step. The FT-IR spectra were recorded using a Bruker Tensor 27 FT-IR spectrometer (Bruker Corporation, Billerica, Massachusetts, United States). The ζ potential and DLS studies were conducted using a ZetaSizer Nano ZS (Malvern Instruments, UK). For carrying out the experiments, the materials studied were suspended in distilled water at a concentration of 1 mg/mL. TGA was performed using a TGA/SDTA 851e Mettler Toledo balance (Mettler Toledo Inc., Schwarzenbach, Switzerland) in an oxidant atmosphere (air, 80 mL/min) with a heating program that consisted of a heating ramp of 10°C/min from 393 K to 1273 K, followed by an isothermal heating step at this temperature for 30 min.

### 2.4 Cytotoxic assay

The human cervical cancer cell line HeLa and human dermal fibroblasts primary cell line HDFn (The American Type Culture Collection, Manassas, VA, United States) were cultivated in Dulbecco’s Modified Eagle’s Medium (high glucose, Sigma-Aldrich, St. Louis, MO, United States) containing 10% fetal bovine serum (Sigma-Aldrich, St. Louis, MO, United States). The cells were cultivated in a monolayer in a humidified atmosphere containing 5% CO_2_ at 37°C. Cells were seeded at 5,000 cells per well in a p96 well-plate for 24 h. Next, the resuspended extract in DMSO with concentration 12,800 μg/mL was serial diluted to 6,400, 3,200, 1,600, 800, 400, and 200 μg/mL concentration levels. Then 1 μL of each concentration level solution was added into the well (final volume of the well was 100 μL) which created levels ranging from 2 to 128 μg/mL in the p96 well-plate. In the case of MSNs, corresponding amounts of nanoparticles (containing equivalent amount of extract) to defined levels were diluted and filled into wells. Doxorubicin (Biosynth, Staad, Switzerland) was used as a positive control, and a medium with DMSO in 1% of the well volume was used as a negative control. The cell viability was determined after 24 h by adding 10 μL Cell Proliferation Reagent—Water-Soluble Tetrazolium Salt 1 into each well. The plates were then incubated for 1 h and measured with spectrophotometer at a wavelength of 450 nm (Multiskan FC Microplate Photometer, Thermo Fisher Scientific, Waltham, MA, United States).

### 2.5 Non-targeted LC-MS analysis

The samples for the non-targeted UPLC-HRAM-MS analysis were prepared by extraction of 50 mg of dry mushroom powder with 0.6 mL of 80% methanol on an orbital laboratory shaker at 210 RPM for 30 min. Subsequently, the mixtures were sonicated for 2 min and centrifuged at 1.12·10^4^ × G for 10 min. The solids (residues) were subsequently reextracted with the identical extraction procedure. Both supernatants were mixed and the extract was stored in −18 °C. For the chemical analysis, the extracts were diluted 100 times in methanol and an internal standard (fluconazole) was added to each sample at a final concentration of 100 ng/mL. To prepare technical blanks, 80% methanol was used instead of the mushroom sample. The analytical measurements were performed using UHPLC-HRAM-MS consisting of a Dionex Ultimate 3,000 (Thermo Fisher Scientific, Waltham, MA, United States) chromatographic system coupled with q-TOF spectrometer Impact II (Bruker Daltonics GmbH and Co. KG, Bremen, Germany). The separation was achieved using a Column Acclaim RSLC 120 C18 (2.2 µm, 2.1 × 100 mm, Thermo Fisher Scientific, Waltham, MA, United States) with pre-column Acquity UPLC BEH C18 (1.7 µm, 2.1 × 5 mm, Waters, MA, United States). The 0.2% formic acid (phase A) and methanol (phase B) were used as mobile phases for gradient elution. The gradient started at 2% of B (0–1 min), then gradually increased to 100% B by the 25th min where it remained until the 35th min, then returned to starting conditions (2% of B) in the 37th min, equilibrated for 10 min with a flow rate of 0.25 mL/min. The column temperature was set at 35°C, and the injection volume was 5 µL. The MS data acquisition was carried out by ESI ionization in both positive and negative modes, with a mass range of 60–1,500 *m*/*z* and a scanning frequency of 2 × 1 Hz (rolling average). The settings of the ion source parameters are given in Supplementary Table S2.

### 2.6 Compound isolation and structure elucidation by NMR and MS^2^

Dry powder from the fruiting body of *G. pfeifferi* (8 g) was extracted in the same way as in Section 2.2, except for dilution in DMSO. Isolation of discriminating compounds for NMR and MS^2^ identification was carried out with the HPLC instrument Dionex Ultimate 3,000 (Thermo Fisher Scientific, Waltham, MA, United States) using RP-100 C18 250 × 4.6 mm column (Watrex, Prague, Czech Republic) with a PDA/UV absorbance detector at wavelengths of 250, 262, and 280 nm. The chromatographic conditions were as follows. The flow rate was 0.5 mL/min, injection volume was 7 mL, and the gradient of the mobile phase B started at 2% (0–1 min) and reached 100% B in the 25th min, which was maintained until the 40th min, then reduced to 2% in 42nd min, and equilibrated for 10 min. The isolated peaks were collected manually into 1.5 mL vials and evaporated under a stream of nitrogen at 37°C. The purity of isolated fractions was confirmed by subsequent analysis on UHPLC-HRAM-MS using the method described previously in Section 2.6. The consequent analysis of fragmentation MS^2^ spectra was performed on the same instrument using collision-induced fragmentation (nitrogen as the collision gas) by MRM mode with a scanning frequency of 1 Hz. The MS^2^ spectra of selected precursor ions (range ±0.5 Da) were recorded at three collision energy levels (15, 25, and 35 eV) except for of 487.3418 *m*/*z* ion (35.45 and 55 eV).

For the NMR structural analysis, the purified fractions were reconstituted in chloroform-*d* with 0.03% tetramethylsilane (TMS). All the chemicals and reagents used were of analytical grade, and deuterated solvents were used such as chloroform-*d* (99.8%) and TMS (>99.5%) (both VWR, Radnor, PA, United States). The acquired 2D NMR spectra consisted of regular proton spectrum, ^13^C attached proton test (APT) experiment, correlation spectroscopy (COSY), heteronuclear multiple bond correlation (HMBC), and heteronuclear single quantum coherence spectroscopy (HSQC). The NMR spectra were recorded on a Bruker Avance III spectrometer equipped with a broad band fluorine observation (BBFO) SmartProbe™ with *z*-axis gradients (Bruker Corporation, Billerica, Massachusetts, United States). The spectrometer operated at the ^1^H NMR frequency of 500.23 MHz and ^13^C frequency of 125 MHz. The spectra were processed with Mnova software version 14.1.0 (Mestrelab Research, A Coruña, Spain). The spectrum was calibrated according to the internal standard (^1^H: TMS = 0 ppm; ^13^C: chloroform = 77 ppm).

### 2.7 Quantitative LC-HRAM-MS analysis of selected compounds

The most important constituents of *G. pfeifferi* characterized and isolated according method described in Section 2.6 were quantified in the extracts of three strains **(GPFE, KZ74, KZ76)** selected for biological testing. The measurement was performed on the UHPLC-HRAM-MS analytical system with an identical separation method and instrumental settings described above (Section 2.5). The analytical standards (ganoderone A, ganoderone B, applanoxidic acid A, applanoxidic acid G) were prepared by HPLC fractionation and characterization of separated peaks described in Section 2.6. Calibration solutions were prepared in methanol at 11 concentration levels in range from 5 ng/mL to 10 μg/mL. For details see Supplementary Material Supplementary Table S3. Samples were measured in triplicate. The peak areas were calculated from extracted chromatograms of [M + H]^+^ ions of the corresponding compounds.

### 2.8 Statistical analysis and data processing

For UHPLC-HRAM-MS analysis, samples of *Ganoderma* strains were at a minimum prepared in triplicate. Prior to the statistical analysis, the raw data were processed using free XCMS online software (ver. 3.7.1). Aligned and extracted features, which are represented by peak areas (PA) of particular *m*/*z* in specific retention time (RT), were filtered using homemade scripts to cut off noise and eliminate multiple ion signals. The featured set was then limited to those with an RT range from 1.5 to 35 min and *m*/*z* values between 100 and 1,000. Features with peak area at least 1·10^5^ (cut-off value) and at least two-fold differences among PA averages within particular mushroom strains were chosen for further statistical evaluation. Two multivariate statistical methods, principal component analysis (PCA) and hierarchical cluster analysis (HCA), were used to investigate the differences among strains and search for unique compounds. All data were normalized by Pareto scaling prior to the analysis. Multivariate statistical analysis was performed using the free online available platform MetaboAnalyst 6.0 (Pang et al., 2022). Quantitative analysis of the selected compounds was performed using TASQ 4.0 software (Bruker Daltonics GmbH and Co. KG, Bremen, Germany).

The results of cytotoxic assays were expressed as percentage of corresponding negative control. Outliers were detected by Grubbs test (a = 0.05) and identified values were excluded from the further analysis. Statistical significance of the differences between positive control and tested samples was determined using two-tailed Student’s t-test (p ≤ 0.01).

## 3 Results

### 3.1 Non-targeted LC-MS and cytotoxicity screening of methanolic extracts

**FIGURE 1:**
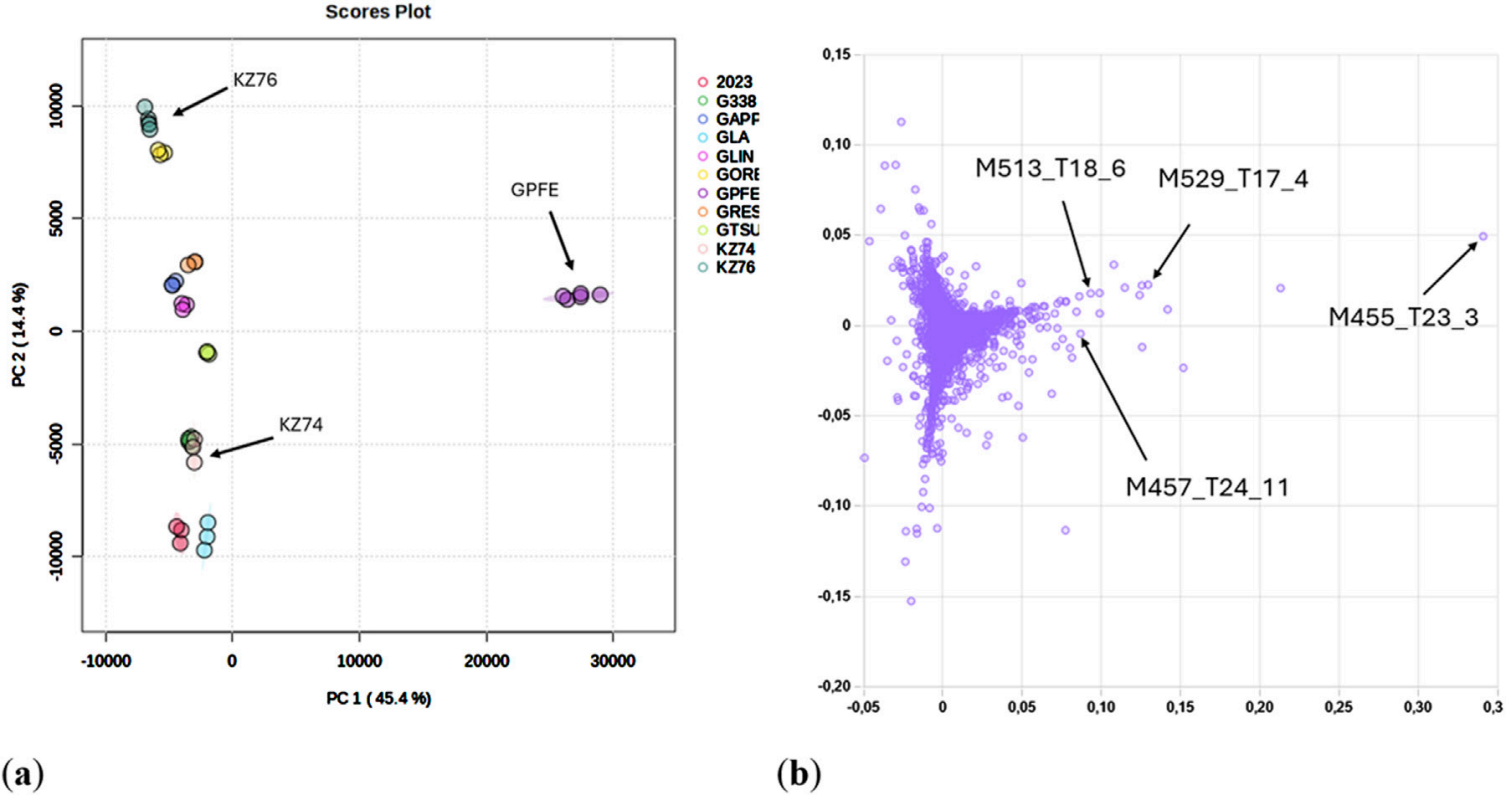
**(A)** PCA score plot of *Ganoderma* samples (Pareto scaling normalization). The representative strains selected for cytotoxic assay are labeled. **(B)** PCA loading plot of all features (peak height ≥1·10^5^ cps) detected in *Ganoderma* samples. The most important features corresponding to [M + H]^+^ ions used for subsequent identification are labeled. *Ganoderma lucidum* var. KZ74 **(KZ74)** *, Ganoderma lucidum* var. KZ76 **(KZ76)** and *Ganoderma pfeifferi* **(GPFE)** *, Ganoderma applanatum* var. 2023 **(2023),** *Ganoderma lucidum* var. 338 **(G338),** *Ganoderma applanatum* **(GAPP),** *Ganoderma. lucidum* var. GA2 **(GLA),** *Ganoderma sichuanense* (linghzi) **(GLIN),** *Ganoderma oregonense* **(GORE),** *Ganoderma resinaceum* **(GRES),** *Ganoderma tsugae* **(GTSU)**.

Results from a liquid chromatography sample screening combined with a mass spectrometric detector and subsequent statistical analysis were obtained and showed in Figure 1. In general, differences in chemical composition among strains of *Ganoderma* genus obtained by nontargeted UPLC-HRAM-MS^2^ analysis and data exploration by multivariate statistical methods PCA and HCA involving 4,995 features showed a significant separation of *G. pfeifferi* (GPFE) from all other compared strains. More than 44% of the total variability in the samples examined, represented by PC1, can be attributed mainly to the different metabolic compositions of *G. pfeifferi*, while the metabolic patterns of the remaining strains were more homogenous. The metabolic distinction of *G. pfeifferi* was also supported by the results of HCA (see Supplementary Material Supplementary Figure S2.), which clearly separated the *G. pfeifferi* cluster from the others. Based on these findings, an extract of GPFE as the exceptional chemotype and two representatives of more metabolically related strains (KZ74 and KZ76) were selected for further chemical characterization and cytotoxic testing.

**FIGURE 2:**
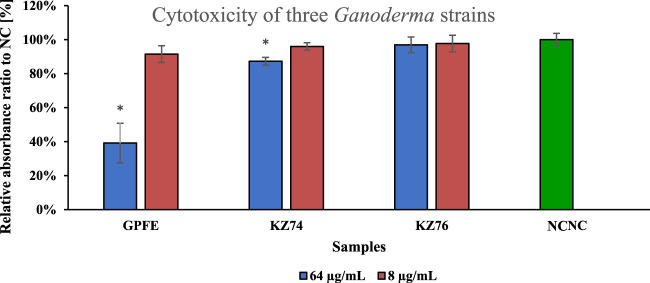
HeLa cytotoxic assay performed at two concentration levels (8 and 64 μg/mL) of the extracts from three *Ganoderma* strains selected on the base of metabolic screening results. Data are represented as means of negative control (**NC**) percentage with standard deviations (n ≥ 3). Values significantly different from the NC are marked with asterisks (t-test, p < 0.01).

The 80% methanolic extracts from the selected strains (GPFE, KZ74, KZ76) resuspended in DMSO were used in the human cervical cancer HeLa cytotoxic assay (Figure 2).

The 8 μg/mL concentration level has not exhibited any apparent cytotoxic activity against HeLa cells, as well as KZ76 extract at 64 μg/mL. The KZ74 extract exhibited slight activity at that concentration level, but the results were close to the NC. Only the GPFE extract exhibited an apparent cytotoxic effect on HeLa cells, and this extract was utilized for further experiments and MSN encapsulation.

### 3.2 Characterization of the main components in *Ganoderma pfeifferi*

**FIGURE 3:**
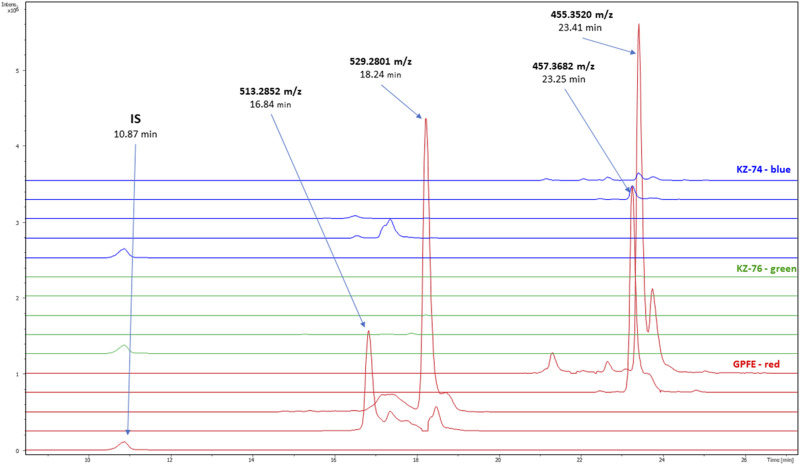
Extracted mass chromatograms of the compounds responsible for the main differences among *Ganoderma sp*. samples in PCA analysis (comparison of three *Ganoderma* species selected for encapsulation and cytotoxic assay); *Ganoderma lucidum* KZ-74—blue, *Ganoderma lucidum* KZ-76—green, *Ganoderma pfeifferi* (GPFE)—red. IS (internal standard—fluconazole, *m*/*z* = 307.1119 [M + H]^+^), ganoderone A (*m*/*z* = 455.3520 [M + H]^+^), ganoderone B (*m*/*z* = 457.3682 [M + H]^+)^ applanoxidic acid A (*m*/*z* = 513.2582 [M + H]^+^), applanoxidic acid G (*m*/*z* = 529.2801 [M + H]^+^).

Based on the results of the HeLa cytotoxic assay, the extract of *G. pfeifferi* was analyzed to identify highly specific compounds for this species that could be responsible for its distinct cytotoxic activity compared to other mushroom extracts tested. The extracted ion chromatograms of these compounds in *Ganoderma* spp. strains used for cytotoxic assays are shown in Figure 3. The most distinctive features are primarily responsible for the separation of *G. pfeifferi* from the remaining *Ganoderma* species in the PCA analysis (see Figure 1B) and were assigned to the probable molecular formulas according to the exact masses of protonated molecules and other adduct ions, isotopic pattern, and retention time. Finally, four compounds with significant signal intensity were chosen that were predominant or occurred exclusively in *G. pfeifferi* extracts and were absent or present only in traces in the other *Ganoderma* samples, including both tested strains of *G. lucidum* KZ74 and KZ76 (Supplementary Figure S3).

**FIGURE 4:**
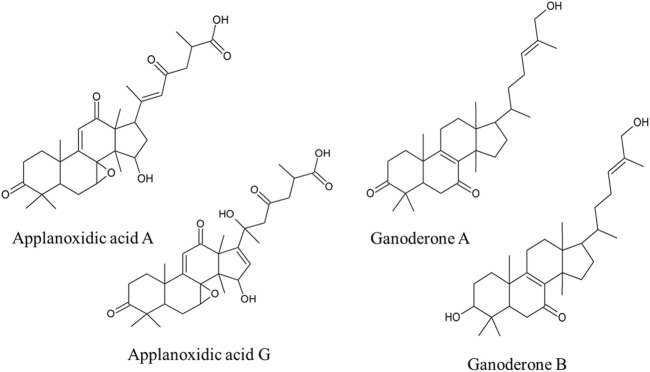
Structures of the most important compounds characteristic for *Ganoderma pfeifferi* extract in comparison with other tested *Ganoderma* strains extracts.

To identify the compounds specific for *G. pfeifferi*, HPLC fractionation was performed. The fractionation process yielded four known compounds: applanoxidic acid A, applanoxidic acid G, ganoderone A, and ganoderone B (Figure 4.). These compounds were confirmed through comparison of their spectroscopic (^1^H and ^1^³C NMR) and spectrometric (MS^2^) data with literature references. The first compound, ganoderone B (lucidadiol), was identified by its molecular formula, C_30_H_48_O_3_, based on HRAM-MS data (observed *m*/*z* 457.3682, calc *m*/*z* 457.3682 for [M + H]^+^), and comparison of its NMR data with those reported by González et al., 1999 (Supplementary Figures S4–S8.). The second compound, identified as ganoderone A, is a ketone derivative of ganoderone B. It shares structural similarities with ganoderone B but lacks the secondary alcohol signal. HRAM-MS data revealed a molecular formula of C₃₀H₄₆O₃ (observed *m/z* 455.3520; calculated *m/z* 455.3525 for [M + H]^+^), and NMR data consistent with Niedermeyer et al., 2005 (Supplementary Figures S9–S13). The remaining two compounds were identified as lanostane-type triterpenes, specifically applanoxidic acids. Applanoxidic acid A, with a molecular formula of C₃₀H₄₀O₇ (observed *m/z* 513.2582; calculated *m/z* 513.2852 for [M + H]^+^), was characterized by the olefinic proton signals (δ_H_ 6.39 and 6.05 ppm, each 1H, s), as reported by Chairul et al., 1991; Niedermeyer et al., 2005 (Supplementary Figures S14–S18). The fourth compound, applanoxidic acid G, was identified with a molecular formula of C₃₀H₄₀O₈ (observed *m/z* 529.2801; calculated *m/z* 529.2801 for [M + H]^+^) and confirmed by comparison of NMR data with Mothana et al., 2003 (Supplementary Figures S19–S23).

The content of the isolated main compounds was determined in dry *G. pfeifferi* fruiting body and varied from 0.04% to 0.21% of dry weight. Ganoderone A was the most abundant metabolite (2.07 ± 0.12 mg/g d. w.) followed by applanoxidic acid G (1.27 ± 0.06 mg/g d. w.), ganoderone B (0.95 ± 0.08 mg/g d. w.) and applanoxidic acid A (0.37 ± 0.01 mg/g d. w.). In both compared *G. lucidum* strains the content of these four compounds was negligible and did not exceed 0.006% of d. w. (Supplementary Table S4).

### 3.3 Encapsulation and nanoparticle characterization

**FIGURE 5:**
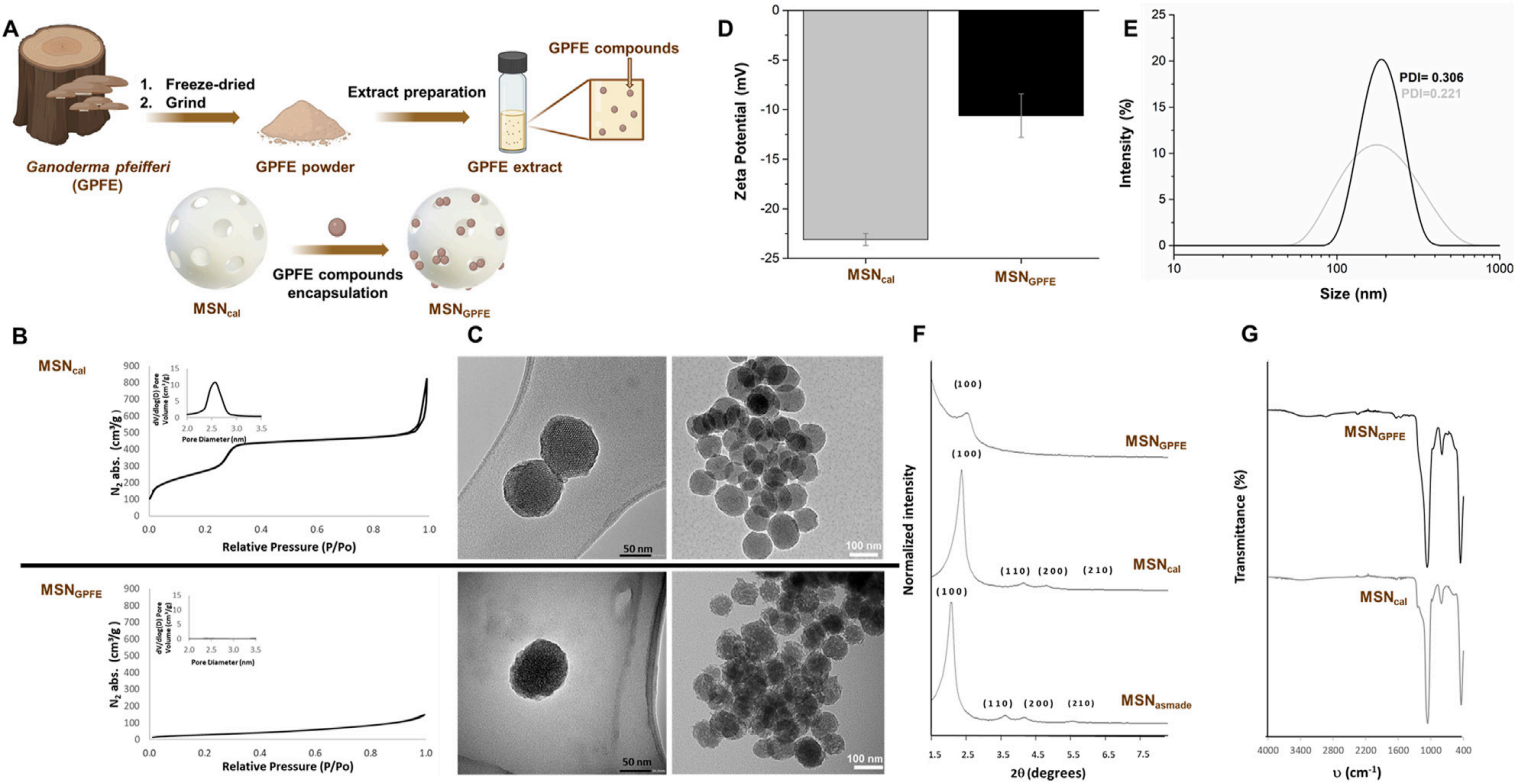
Encapsulation process and nanoparticles characterization. **(A)**, **(B)** nitrogen adsorption-desorption isotherms, **(C)** TEM images (MSNcal top and MSN_GPFE_ bottom at different magnifications), **(D)** zeta-potential plot with its errors, **(E)** DLS plot with PDI, **(F)** powder X-ray diffractograms, **(G)** FT-IR plot.

The workflow of encapsulation is graphically described in Figure 5A. Mesoporous silica nanoparticles (MSNs) type MCM-41 were synthesized following the atrane route conducted as described in the Materials and Methods Section. Then, the mesopores of the silica nanoparticles were loaded with the *G. pfeifferi* methanolic extract, obtaining **MSN**_**GPFE.**_ The MSNs and **MSN**_**GPFE**_ were characterized with the following standard methods such as TEM, N_2_ adsorption-desorption isotherms, PXRD, FT-IR, zeta (ζ) potential, DLS and TGA.

N_2_ adsorption-desorption isotherms were performed for the MSNs in order to study the specific surface area, pore volume, and pore size (Figure 5B.). The N_2_ adsorption-desorption isotherms of the calcinated MSNs showed a type IV isotherm, typical of mesoporous silica materials, in which two adsorption steps can be observed (Figure 5B upper chart). The first adsorption step at P/P_0_ below 0.3 is attributable to the condensation of nitrogen within the empty pores. Furthermore, the narrow pore size distribution (inset in Figure 5B upper chart) and the absence of the hysteresis loop in this range suggest the presence of uniform cylindrical mesopores. Conversely, in the N_2_ adsorption-desorption isotherms of **MSN**_**GPFE**_, a significant decrease in the adsorbed N_2_ volume and surface area is observed due to the loading inside the mesopores (Figure 5B lower chart and inset Figure 5B lower chart). The specific areas of the total surface area were calculated using the BET model. Additionally, to calculate the pore size and volume, the BJH model was applied to the adsorption band of the isotherm for P/P_0_ < 0.6. All data are summarized in Supplementary Table S5.

TEM images of **MSN**_**cal**_ and **MSN**_**GPFE**_ were also acquired (Figure 5C.). As seen in Figure 5C., the TEM analysis of the MSNs showed that nanoparticles exhibited a spherical geometry with a diameter of about 100 nm. Also, in TEM images, typical pores of the MSNs MCM-41 are present. However, in **MSN**_**GPFE,**_ these channels are no longer visible after the loading step, likely due to the contrast difference caused by the filling of the pores. Hydrodynamic size and ζ potential of different MSNs were also used to follow the loading process (Figure 5D.). For the zeta potential study, **MSN**_**cal**_ shows a value of −23.09 mV due to the presence of silanol groups. After the extract loading, the corresponding solid displays a less negative surface potential due to the silanol group’s pore blocking, achieving a −10.6 mV. For the hydrodynamic size (DLS), Figure 5E and Supplementary Table S6 also show how the hydrodynamic size changes from 169.4 nm for **MSN**_**cal**_ to 216.6 nm for **MSN**_**GPFE**_, and PDI values changes from 0.221 for **MSN**_**cal**_ to 0.306 for **MSN**_**GPFE**_. Finally, thermogravimetric studies were carried out to obtain the quantification of the cargo (Table 1).

**TABLE 1:** Organic contents of extract loaded into nanoparticles (%).

Residual solvent (%)	Organic matter (%)	Silica (%)	Organic matter to silica ratio (%)
3.91	25.05	71.05	35.26

Figure 5F shows powder X-ray diffraction patterns at low angles (1.5 < 2θ < 7) of the MSN before and after calcination and the final loaded nanoparticles. The PXRD patterns of the “as made” showed four main peaks at low angles that can be indexed as the (100), (110), (200), and (210) Bragg reflections, which are characteristic of hexagonally ordered mesoporous nanoparticles. A shift of the 2 θ values of these reflections to the right was observed after the calcination process of the MSNs. This is due to the removal of the surfactant and condensation of the silanol groups during the calcination process and shrinkage of the mesostructure. In the final MSN loaded with the extract, the peaks (110), (200), and (210) in their PXRD patterns disappeared due to a contrast reduction caused by the loading procedure. Nevertheless, preserving the (100) reflection in their PXRD patterns indicated that the mesoporous structure had been preserved.

Furthermore, infrared spectroscopy was employed to ensure the correct encapsulation of extract inside the pores. FT-IR spectra of all the solids within the 4,000–400 cm^−1^ wavelength range are shown in Figure 5G. Solids present the characteristic bands of the silica matrix (Figure 5G lower chart); the stretching of the Si-O-Si bonds above 1,060 cm^−1^, the bending of the Si-O bond around 790 cm^−1^, and a rocking signal at 450 cm^−1^, in addition to bands related to vibrations of water molecules (3,420 and 1,620 cm^−1^). Smaller bands related to the loading process can be observed with additional changes. Thus, the loaded MSNs spectrum (Figure 5G upper chart) shows the appearance of bands around 1,550–1700 cm^−1^, and bands around 2,500 and 2,800 cm^−1^ attributed to the extract loading are also observed. Finally, a broad band was found above 3,400 cm^−1^, attributable to the water molecules adsorbed.

### 3.4 Cytotoxicity of the encapsulated *Ganoderma pfeifferi* extracts

In the second assay, two forms of the GPFE extract were used in the HeLa cytotoxic assay - the nano-encapsulated extract (**MSN**_**GPFE**_ **)** and the free extract (Figure 3.). The amount of nanoparticles added to the well volume was calculated with respect to the organic matter content in nanoparticles (Table 1).

TGA can be seen in Table 1, which allowed us to calculate an organic matter content of mushroom extract loaded into MSNs of 25.05%.

The two forms were compared to determine their effectiveness. The efficiency of encapsulated extracts (**MSN**_**GPFE**_ **)** against HeLa cells was found to be similar to that of free extracts, but there was no substantial enhancement. In the non-tumorigenic human fibroblast cell line the situation was different. This cell line was generally less susceptible to the GPFE extract and encapsulation completely diminished its toxicity even in the highest concentration tested. The IC_50_ for resuspended dimethyl sulfoxide extract was assessed and found to be 20.5 μg/mL, and for MSNs, it was 16.6 μg/mL, which indicated that its efficacy increased by 23.5%. As a positive control, doxorubicin was used at concentration level of 10 μg/mL.

## 4 Discussion

The aim of this study was to attempt to identify low-molecular-weight biologically active compounds from *Ganoderma* spp., which could be potentially responsible for their cytotoxicity, using non-targeted screening combined with *in vitro* biological activity testing. *Ganoderma pfeifferi* was found to contain the most distinct metabolic profile in non-targeted analysis. This species found exclusively in Europe (Mothana et al., 2000) had only a limited scientific focus on phytochemistry and medicinal potential (Lindequist, Jülich, and Witt, 2015). A previous study reported the isolation and characterization of three sterols and three triterpenoids from *G. pfeifferi,* which were tested in NO production activity evaluated by RAW 264.7 cells. Among selected compounds, only ergosterol significantly decreased NO production (Kuo et al., 2016). No study of the effects of *G. pfeifferi* constituents on HeLa cervical cancer cells used in our work has been published yet. In contrast, extensive studies have dealt with another species of *Ganoderma* genus, *G. lucidum*, that has been studied for its antiproliferative effects on human cancer cells, including HeLa cell line (Zhu et al., 2000; Yue et al., 2010; Liu and Zhong, 2011; Ru-Ming, Ying-Bo, and Jian-Jiang, 2012). For example, a study conducted with ethanol extract and the silica gel purified ethanol extract of *G. lucidum* sporoderm-broken or nonbroken spores revealed an inhibition of growth in a dose-dependent manner (48 h incubation, at different concentrations 0–4.8 mg/mL). IC_50_ for crude extract was determined equal to 4,460 μg/mL and purified extract equal to 750 μg/mL, which suggests the purified extract is almost six times as potent as the crude one (Zhu et al., 2000). A recent study investigated the cytotoxic effect of a crude extract of *Ganoderma applanatum in vitro*. The IC_50_ value was determined to be 1,550 ± 10 μg/mL concentration against HeLa cancer cells for the ethanol extract, and for 2(5H)-furanone identified as a likely responsible compound, the IC_50_ decreased to 1.99 ± 0.01 μg/mL (Kiddane et al., 2022). The present study focused on solvents with similar polarity, specifically, methanol. The IC_50_ value determined in the present study was 20.5 μg/mL. These findings suggest that the *G. pfeifferi* extract examined in present research exhibited greater potency compared to the purified *G. lucidum* ethanol extract. It might explain the low activity of 2 *G. lucidum* at these concentration levels. In another study, where individual ganoderic acids were assessed against HeLa cells, the resulting IC_50_ values for individual ganoderic acids ranged from 15.1 to 20.3 μM, which, recalculated with respect to the molar mass of individual compound, ranges between 8.7 and 11.4 μg/mL (Yue et al., 2010). Comparing present study to this investigation, results suggest that the extract was about half as powerful in terms of IC_50_.

Regarding the cytotoxicity of the four compounds isolated from *G. pfeifferi* in this study, only the activity of applanoxidic acid A against the HL-60 cell line has been reported (Galappaththi et al., 2022). To our knowledge, no valid data on their activity against cancer cells have been published for applanoxidic acid G, ganoderone B, and ganoderone-A.

The use of the *Ganoderma* spp. extracts tested in our study and the substances isolated from them with potential biological activity in clinical applications or as food supplements depends on their toxicity to the human organism. Due to the chemical nature of the main components of the genus, mainly belonging to triterpenoid derivatives, the potential hepatotoxicity of the *Ganoderma* spp., especially widely used *G. lucidum* (Reishi), is most often discussed in several case studies (Yuen et al., 2004; Khan et al., 2023). However, almost all of these cases were associated with high doses, the simultaneous use of *Ganoderma* products with other substances or certain pathological conditions and are recorded very rarely (Guedikian et al., 2023; Wanmuang et al., 2007). On the contrary, the vast majority of publications regarding the effect of *Ganoderma* on the liver report their hepatoprotective properties (Ahmad et al., 2021; Johra et al., 2023). The lack of toxicity of lower doses of the *Ganoderma* and its good tolerability have been confirmed in a number of preclinical and clinical studie (Noguchi et al., 2008; Ahmad et al., 2021). Regarding *G. pfeifferi* and its isolated compounds involved in our study, to the best of our knowledge there is no relevant scientific information about its potential toxicity in the literature. Nevertheless, based on above-mentioned case studies reporting serious health complications associated with the use of *Ganoderma* spp. products, potential toxic effects should be further investigated.

A notable and novel aspect of the present study involved the rare approach with the use of loaded extract in nanoparticles. In this research, the mesoporous silica nanoparticles (MSNs) were synthesized and characterized both empty and loaded with mushroom methanolic extract. In traditional medicine, dried powdered fungal material is commonly used. In our case, obtaining the extracts was a critical point for the nanoencapsulation process since much of the powder generated by the freeze-drying process is organic matter from the *Ganoderma* matrix itself, which does not show anticancer properties. For the loading process, a complete dissolution of the compounds to be encapsulated is necessary since a good loading cycle is not usually completed in terms of efficiency and yield in heterogeneous solutions and may remain adhered to the surface of the nanoparticles and not nano-encapsulated in their pores, which would complicate the effective transport of the compound. As described in the literature, various types of mesoporous silicas have unique properties that allow them a wide range of guest species (Ogawa, 2017). These particles have a mesostructured pore size, nano-micro particle size and high loading capacity, making them ideal for a wide range of host-guest systems, including the one developed in this research. The present study utilized the MCM-41 type of MSN, which is one of the most widely used and analyzed nanoparticles. In all of the examples of MCM-41 type use, standard materials and inorganic chemistry characterization techniques were used to analyze the particles. Characterization is necessary not only to ensure the proper synthesis and loading of MSN materials but also to monitor the synthesis steps and understand the mechanism. The most common characterization techniques include powder X-ray diffraction, N_2_ adsorption-desorption, transmission electron microscopy, dynamic light scattering, thermogravimetric analysis, Fourier-transform infrared spectroscopy, elemental analysis (EA), and UV-visible and fluorescence spectroscopy. All of these techniques have been used in present research, showing synthesized materials have the characteristic structural parameters for ordered mesoporous materials and the well-defined structures (Vicente et al., 2024).

Specifically, the presence of the mesoporous structure in the final solid is also observed from the TEM analysis, in which the typical disordered hexagonal porosity of the silica mesoporous matrix can be observed. As it was mentioned above, in **MSN**_**GPFE,**_ these channels are no longer visible after the loading step, most likely due to the contrast difference caused by the filling of the pores. The N_2_ adsorption-desorption isotherms also support the demonstration of the proper loading procedure. The high amount of organic matter related to the extract GPFE sample in the final solid **MSN**_**GPFE**_ blocks the adsorption of gas molecules, so the registered curve is completely flat when compared with the one of the empty starting material, where the absence of appreciable mesoporosity is observed. Furthermore, the PXRD patterns of all solids show the characteristic hexagonally ordered mesoporous structure for this type of silica nanoparticles (Beck J. S. et al., 1992; Kresge et al., 1992). Moreover, the reduction of contrast of the final solid **MSN**_**GPFE**_ PXRD pattern is due to the filling of the pore voids with the GPFE sample. Nevertheless, the clear presence of the (100) peak in this pattern suggests that the loading process with the extract has not substantially damaged the mesoporous nanoparticle. Infrared spectroscopy is also a suitable technique for following the loading process leading to solid **MSN**_**GPFE**_. The specific presence of bands around 1,550–1700 cm^−1^ and around 2,500 and 2,800 cm^−1^ are attributed to the GPFE extract and linked with the content of the compounds, such as ganoderone A, ganoderone B, applanoxidic acid A, or applanoxidic acid G. The loading process was also followed by the ζ potential, changing the starting negative ζ value after loading since the active groups of the GPFE react with the silanolates of the MSN empty. Dynamic light scattering measurements revealed a hydrodynamic diameter for the starting MSNs and final **MSN**_**GPFE**_ nanoparticles of 169.4 nm and 216.6 nm, respectively (Galiana et al., 2020). The increase in the hydrodynamic diameter of **MSN**_**GPFE**_ is consistent with the loading of the nanoparticles with GPFE. And finally, the TGA values for **MSN**_**GPF**_ nanoparticles, indicated 25.05 mg of GPFE per Gram of **MSN**_**GPFE**_ (Poyatos-Racionero et al., 2021; Galiana et al., 2020). In this scenario, the successful loading procedure of the GPFE into the 2 nm mesopores of the MSNs was demonstrated.

**FIGURE 6:**
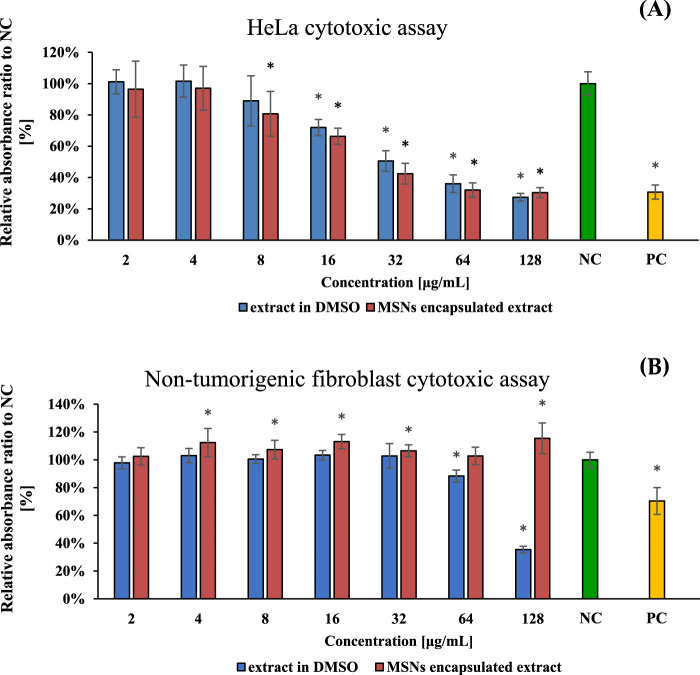
HeLa **(A)** and non-tumorigenic fibroblast **(B)** cytotoxic assay at serial concentrations of *Ganoderma pfeifferi* extract dissolved in dimethyl sulfoxide (DMSO) – blue–and encapsulated in MSNs nanoparticles (=MSN_GPFE_) - orange. **NC**—negative control (green), 1% (*v*/*v*) of DMSO and dispersed empty MSNs. **PC**—positive control (yellow), doxorubicin (10 μg/mL) and 1% (*v*/*v*) of DMSO. Data are represented as means of NC percentage with standard deviations (n ≥ 6). Values significantly different from the NC are marked with asterisks (t-test, p < 0.01).

Even though the encapsulated extract (**MSN**_**GPFE**_) did not show substantial enhancement of the cytotoxicity against cancer cells, it significantly decreased the toxicity against healthy fibroblasts (Figure 6B.). In addition, the technological properties are different and favour further functionalization for target delivery of the extracts, which is a common aim of these technologies (Vallet-Regi et al., 2001). A similar study on complex mushroom extract has not yet been conducted. Studies reporting effect of encapsulation of natural products on their cytotoxic activity are mainly focused on plant extracts or isolated bioactive compounds (Priyanka et al., 2019; Guillén-Meléndez et al., 2024). The effect of encapsulation on biological activity was demonstrated in a study comparing camptothecin, a cytotoxic alkaloid from the bark of the *Camptotheca* tree used in traditional Chinese medicine, encapsulated in MCM-41 particles and applied in free form against HepG2 cancer cells. The results showed that encapsulation increased the cytotoxicity of camptothecin by 9.2% at the same concentration (Priyanka et al., 2019). A similar work, where cytotoxic plant alkaloid ellipticine was encapsulated in MCM-41, which improved the efficacy against HeLa cancer cells compared to free ellipticine by 129% on an equivalent concentration level and increased HeLa cell death at a 50 μM concentration (cell viability is 2.58% ± 0.35%, compared to free form 12.8% ± 0.64%). The mechanism of the NP effect was explained as loaded NPs internalized in the cancer cells and localized temporally into the nucleus (Koninti et al., 2016). Although the present study did not demonstrate such a dramatic difference in IC_50_ between MSNs-encapsulated (16.6 μg/mL) and free *G. pfeifferi* extract (20.5 μg/mL), an encapsulation increased its cytotoxicity by 23.5%. On the other hand, the use of nanoparticle may as well decrease the cytotoxic effect as it was observed in a study, where loading curcumin into MCM-41 had a counterproductive effect on its activity against the squamous cell carcinoma SCC25 cell line in cytotoxic assay. The most potent form of curcumin was observed to be the free form in DMSO (Jambhrunkar et al., 2014). A study involving the use of combined nanoparticles consisting of chitosan, quantum carbon dots, and aptamers. The nanoparticles were tested for delivery of the model cytotoxic drugs: 5-fluorouracil and combination of ganoderic acid and 5-fluorouracil to MCF-7 breast cancer. The results of combined ganoderic acid, 5-fluorouracil in form of nanoparticle with aptamers demonstrated the greatest cytotoxic effect, followed by the combination without aptamers. The least cytotoxic effect showed free 5-fluorouracil (Mazandarani et al., 2023).

Despite the fact that the cytotoxic efficacy of GPFE extract against HeLa cells was only slightly improved in our study, the key point is that the loaded extract is able to be released from MSN nanoparticles and effectively act against cancer cells. Compared to the free GPFE extract, the encapsulated form (**MSN**_**GPFE**_) was significantly less toxic to non-tumorigenic fibroblast cells at concentrations exceeding 64 μM (Figure 6B.), while for the HeLa tumor line, the toxicity of the encapsulated form was still considerably high at the same concentrations (Figure 6A.). Thus, the application of complex *Ganoderma* spp. extract loaded in NPs represents a novel approach enhancing its bioactivity.

Questions on the toxicity of the MSNs could be raised as well, because the immunogenicity and toxicity derived from the bioaccumulation of this type of inorganic carrier is one of the most critical challenges for their clinical translation. So far, different contributions describe the mechanism of MSN elimination and confirm that MSNs are effectively eliminated from the body regardless of the route of administration, dose, shape, size, and surface properties (Yuan et al., 2019; Li et al., 2020). Several data demonstrate that MSNs are biocompatible in animal models, but studies have focused more on their application than on assessing their toxicity. Therefore, one of the main obstacles to employing MSNs as a delivery strategy for biomedical applications is the lack of knowledge about the long-term safety of nanomaterials, which needs to be addressed. Nevertheless, significant progress has been made in the development of new biodegradable and more clearable MSNs with reduced toxicity while retaining useful therapeutic or imaging functions (Lérida-Viso et al., 2023).

## 5 Conclusion

In this study, the methanolic extracts of 11 strains of 7 species of wood decay mushrooms genus *Ganoderma* spp. were compared in non-targeted study using UHPLC-HRAM-MS analysis. Based on their metabolomics profile, three representatives (*Ganoderma lucidum* var. KZ74, *G. lucidum* var. KZ76, and *G. pfeifferi*) were selected and tested for their antiproliferative properties against HeLa cancer cells. Mesoporous silica nanoparticles were utilized to establish the increase in efficacy with encapsulation. Extracts were fractioned and analyzed with UHPLC-HRAM-MS^2^ and NMR. Low molecular bioactive compounds associated with the *Ganoderma* genus were identified. The solid extract residues of evaporated raw methanolic extracts were resuspended in dimethyl sulfoxide and subsequently tested in the HeLa cytotoxic assay. The bioactivity revealed that the only active sample was *G. pfeifferi* (GPFE), with *G. lucidum* having not demonstrated any significant activity. MCM-41 mesoporous nanoparticles were synthesized and characterized. The nanoparticles were loaded with the extract and used in the HeLa cytotoxic assay to compare the efficacy of the free form versus the encapsulated form of the extract. The study’s results suggest that the encapsulated form **MSN**_**GPFE**_ also demonstrated activity against HeLa cells. The efficacy of the encapsulated form increased by about 23.5% (in the case of IC_50_ comparison), and the extract exhibited promising results at a concentration of 128 μg/mL compared to PC. All the results indicate, that both *G. pfeifferi* and the encapsulation strategy of their bioactive components worth further research. Future studies should focus on isolation and identification of the most potent constituents and subsequent steps of their cytotoxic research, like evaluation of mechanisms of their action. Also, the beneficial effects of their encapsulation to MSNs should be further investigated.

## Data Availability

The datasets presented in this study can be found in online repositories. The names of the repository/repositories and accession number(s) can be found in the article/Supplementary Material.

## Glossary

APT: attached proton test
BET: Brunauer-Emmett-Teller theory
BJH: Barrett-Joyner-Halenda Model
COSY: corelated spectroscopy
cps: counted per second
d.w.: dry weight
DLS: dynamic light scattering
DMSO: dimethyl sulfoxide
ESI: electrospray ionization
GPFE: *Ganoderma pfeifferi*
HCA: hierarchical cluster analysis
HDFn: primary cell line of neonatal human dermal fibroblasts
HeLa: type of cervical cancer cell of Henrietta Lacks
HMBC: heteronuclear multiple bond correlation
HSQC: heteronuclear single quantum coherence
**IC**_**50**_: half maximal inhibitory concentration
KZ74: sample, strain of *Ganoderma lucidum*
KZ76: sample, strain of *Ganoderma lucidum*
LC-MS: liquid chromatography couple with mass spectrometry detection
MCM-41: mobil composition of matter No. 41, type of nanoparticle
**MSN**_**cal**_: calcinated mesoporous silica nanoparticles
**MSN**_**GPFE**_: mesoporous silica nanoparticles loaded with *Ganoderma pfeifferi* extract
MSNs: mesoporous silica nanoparticles
NC: negative control
NO: nitric oxide
NPs: nanoparticles
PC: positive control
PCA: principal component analysis
PXRD: powder X-ray diffraction
TCM: trichlormethane
TEM: transmission electron microscopy
TGA: thermogravimetric analysis
TMS: trimethylsilane
