# Supplementary material for: Chemical characterization and encapsulation of Ganoderma pfeifferi extract with cytotoxic properties
Source: Front Pharmacol. 2025 Jan 23;16:1526502. doi: 10.3389/fphar.2025.1526502 (PMC11799868; doi:10.3389/fphar.2025.1526502)
Supplement: Supplementary file 1 [file DataSheet1.docx]

Supplementary Material


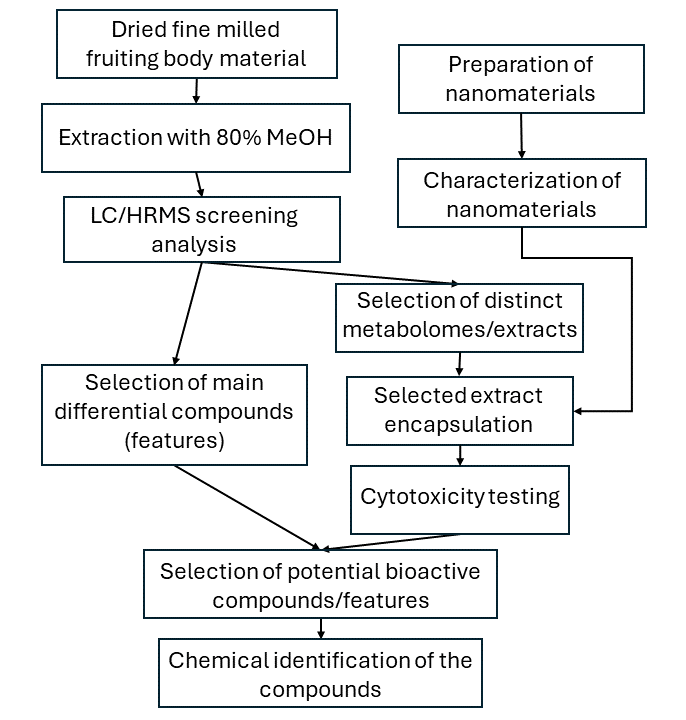


**Figure S1.** Flowchart of the experimental design

**Table S1.** The preliminary results of yield of extraction of mushrooms powder samples with 3 types of solvents (1 g of sample weighed) (n=2)

| **Strain** | **Average yield by solvent type (%)** | | |
| --- | --- | --- | --- |
|  | **80% MeOH^a^** | **trichlormethane^b^** | **Hexane^b^** |
| *Ganoderma pfeifferi* (GPFE) | 10.36 | 5.18 | 1.22 |
| *Ganoderma lucidum* var. KZ74 (KZ74) | 5.42 | 3.71 | 0.93 |
| *Ganoderma lucidum* var. KZ76 (KZ76) | 16.22 | 3.67 | 1.50 |

a) 12 mL of solvent added to 1 g of sample, extraction and reextraction with 12 mL of the same solvent, both extracts mixed and evaporated

b) 2 mL of purified demineralized water (for soaking the powder material for supernatants separation) and 10 mL of the solvent, extraction and reextraction with 10 mL of the same solvent, both extracts mixed and evaporated

| **Ion source settings** | Ion source | ESI |
| --- | --- | --- |
|  | Ionization mode | Positive |
|  | End plate offset | 500 V |
|  | Capillary voltage | 2500 V |
|  | Nebulizer gas | 2.0 Bar |
|  | Dry gas | 5.0 L/min |
|  | Dry temperature | 300 °C |
| **Acquisition settings** | Mass range | 60–1500 *m*/*z* |
|  | Scan rate | 1 Hz |
|  | Resolution | > 60 000 |
| **Chromatography conditions** | Column | Acclaim RSLC 120 C18, 2.2 mm, 2.1 x 100 mm |
|  | Precolumn | VanGuard^TM^ ACQUITY UPLC® BEH C18, 1.7 mm, 2.1 × 5 mm |
|  | Flow rate | 0.25 mL/min |
|  | Column temperature | 35 °C |
|  | Mobile phases | A) 0.2% formic acid |
|  |  | B) MeOH |
| **Gradient** | \| T [min] \| 0 \| 1 \| 25 \| 35 \| 37 \| 47 \| \| --- \| --- \| --- \| --- \| --- \| --- \| --- \| \| B [%] \| 2 \| 2 \| 100 \| 100 \| 2 \| 2 \| | |

**Table S2.** Detailed HRAM-MS^2^ and chromatography measurement conditions

**Table S3. Calibration parameters for quantitative LC-HRAM-MS analysis of the four selected compounds**

| **Compound** | ***m*/*z* [M+H]^+^** | **RT [min]** | **Equation** | **R^2^** | **LOD^*^ [μg/g]** | **LOQ^**^ [μg/g]** | **Accuracy [%]** |
| --- | --- | --- | --- | --- | --- | --- | --- |
| applanoxidic acid A | 513.2828 | 17.29 | y = 26294·x + 81180 | 0.9997 | 189.72 | 574.91 | 4.42 |
| applanoxidic acid G | 529.2783 | 18.74 | y = 23550·x + 141714 | 0.9994 | 229.98 | 696.92 | 6.28 |
| ganoderone A | 455.3502 | 23.73 | y = 17470·x + 93349 | 0.9992 | 127.39 | 386.04 | 3.72 |
| ganoderone B | 457.3682 | 23.25 | y = 21449·x + 118750 | 0.9991 |  |  | 5.98 |

* LOD was estimated as LOD = 3.33 · SDb/A, where SE is the standard deviation of the intercept, A is the slope of the calibration line

**LOQ was estimated as LOQ = 10 · SDb/A, where SE is the standard deviation of the intercept, A is the slope of the calibration line


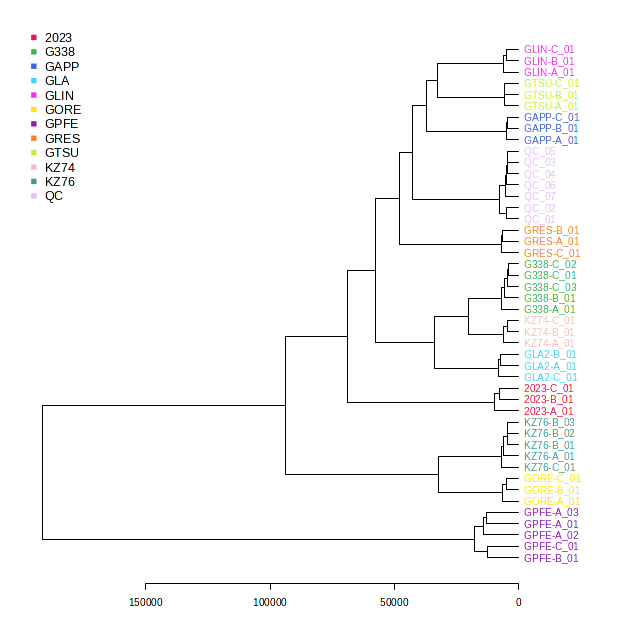
 **Figure S2.** Hierarchical clustering according to metabolic profiles of compared *Ganoderma* genotypes


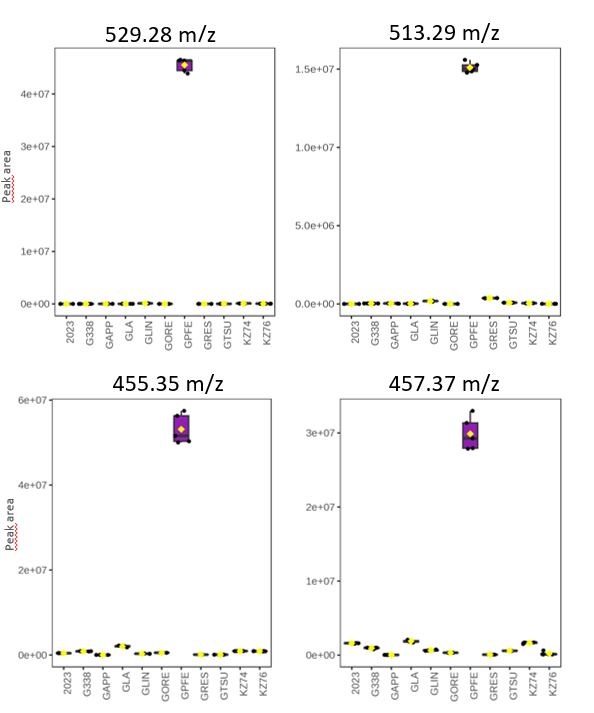

**Figure S3.** Box plot of selected *Ganoderma* strains with the four features mentioned in the Results: applanoxidic acid G (529.28 [M+H]^+^), applanoxidic acid A (513.29 [M+H]^+^), ganoderone A (455.35 [M+H]^+^), ganoderone B (lucidadiol) (457.37 [M+H]^+^).

**Fragmentation spectra of the most important compounds found in *G. pfeifferi***
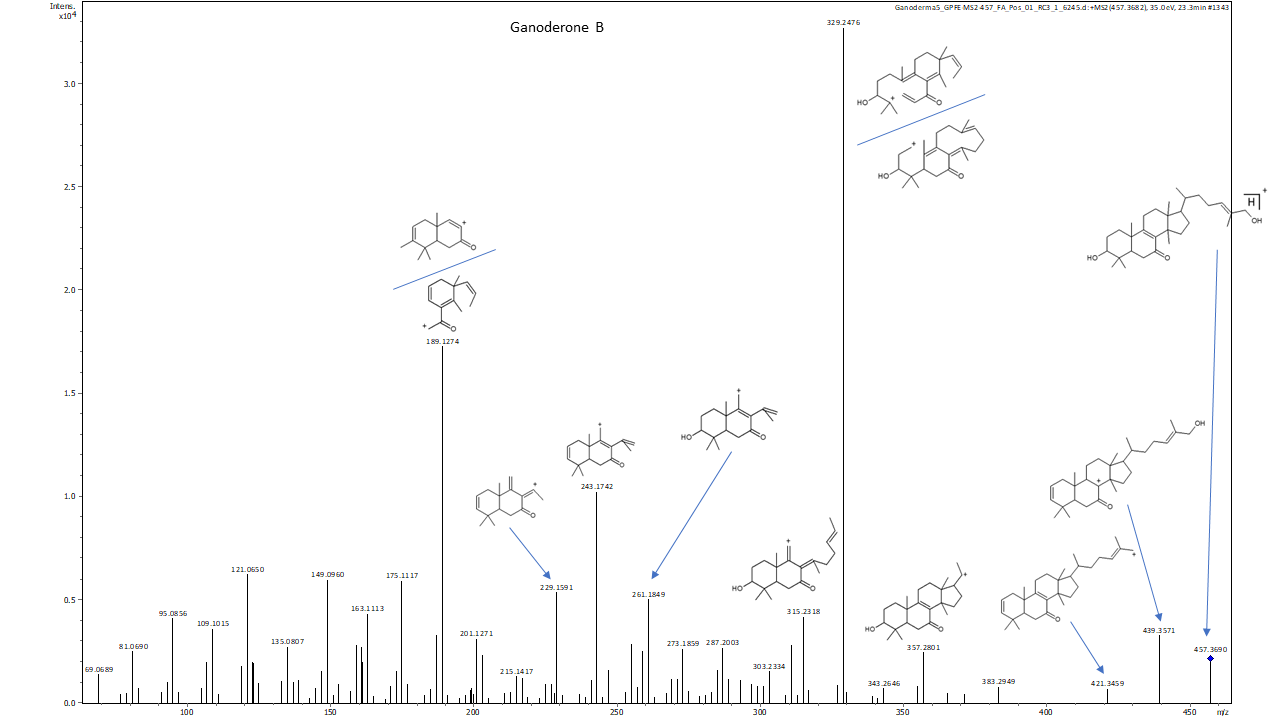

**Figure S4.** Annotated HRAM-MS^2^ spectrum of ganoderone B (lucidadiol) (*m*/*z* = 457.37 [M+H]^+^, RT = 23.25 min).


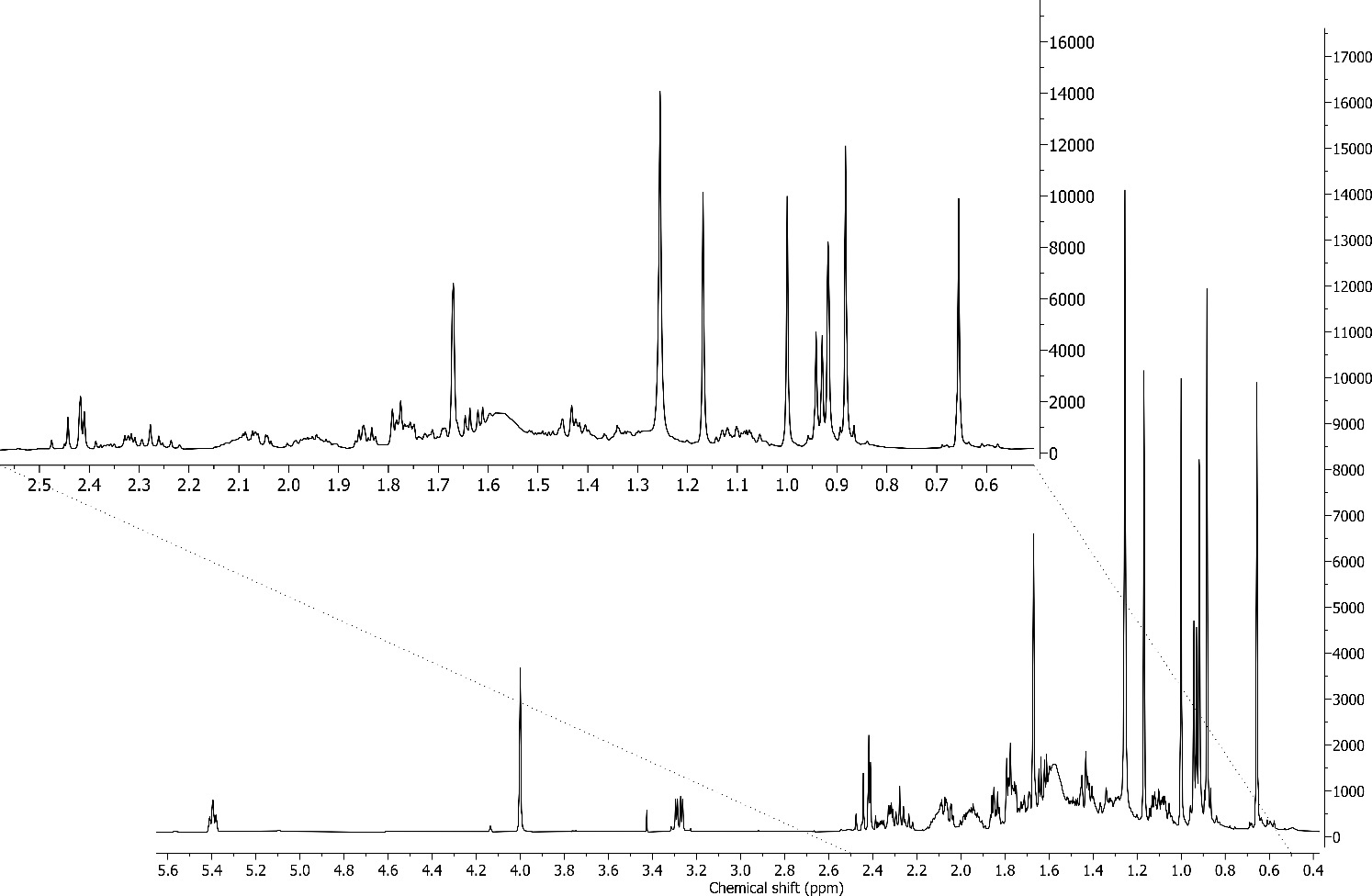


**Figure S5** ^1^H spectrum of purified ganoderone B in CDCl_3_ (500 MHz).


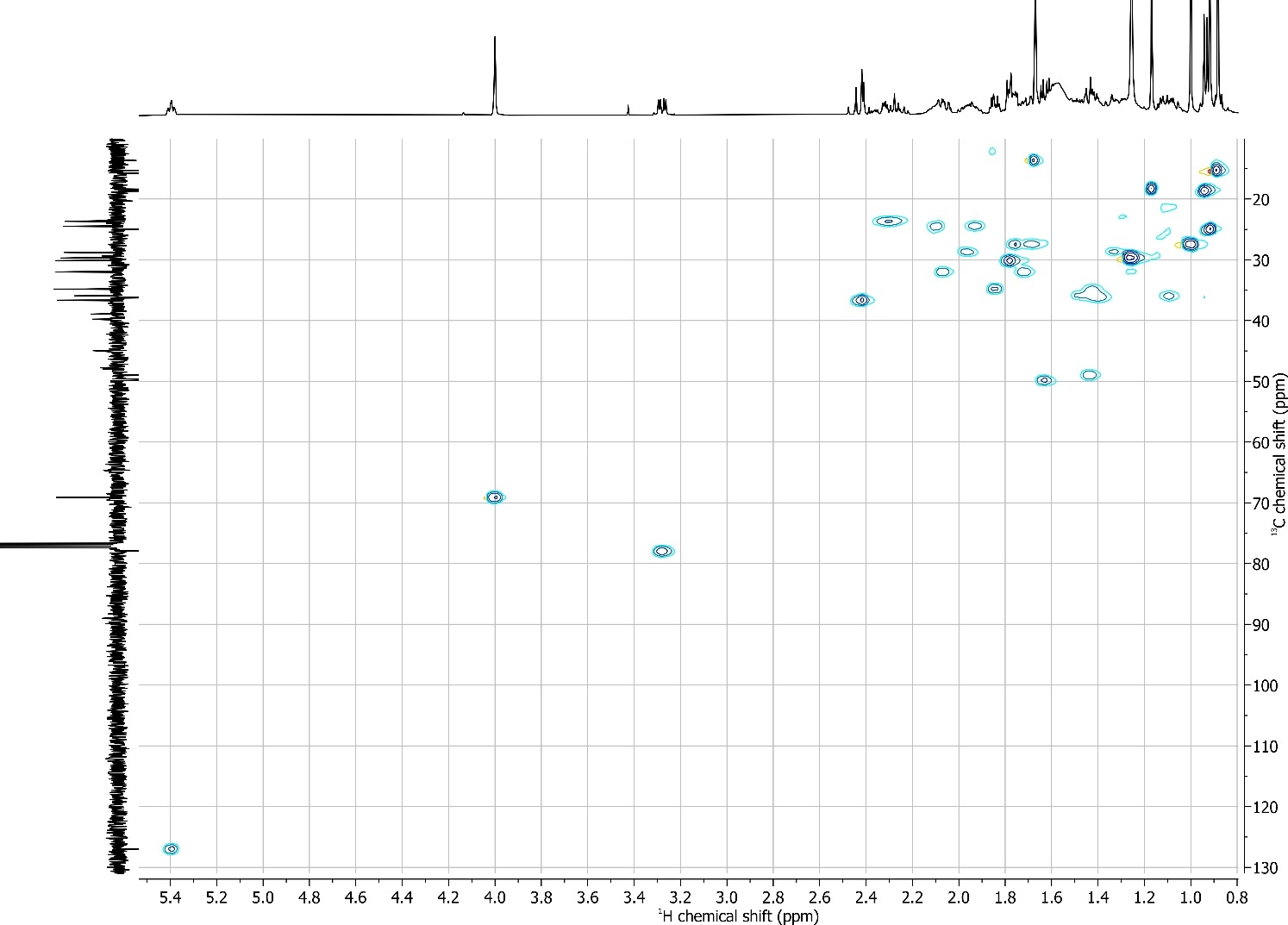


**Figure S6** HSQC spectrum of purified ganoderone B in CDCl_3_ (^1^H 500 MHz, ^13^C 125 MHz).


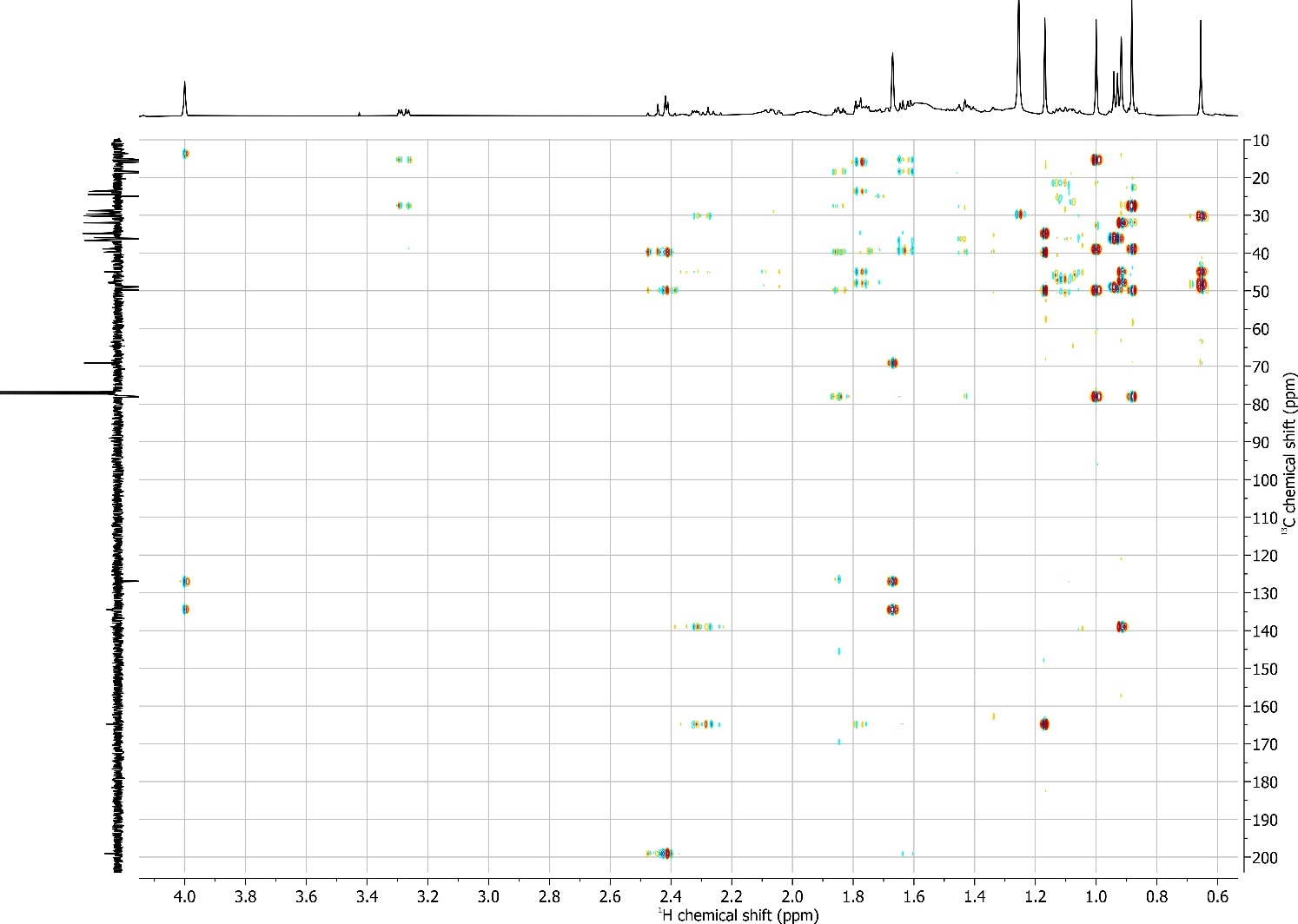


**Figure S7** HMBC spectrum of purified ganoderone B in CDCl_3_ (^1^H 500 MHz, ^13^C 125 MHz).


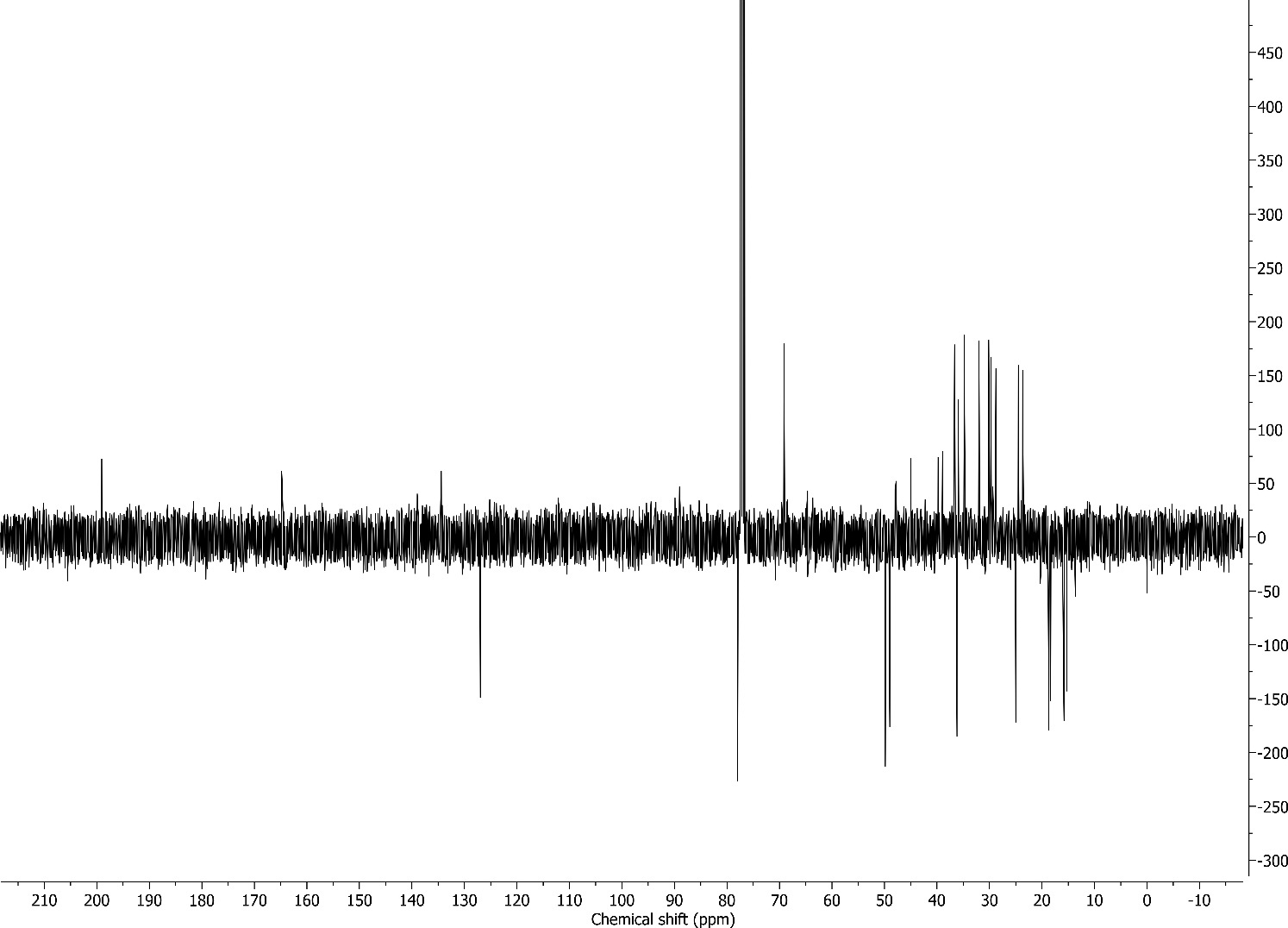


**Figure S8** ^13^C APT spectrum of purified ganoderone B in CDCl_3_ (125 MHz).


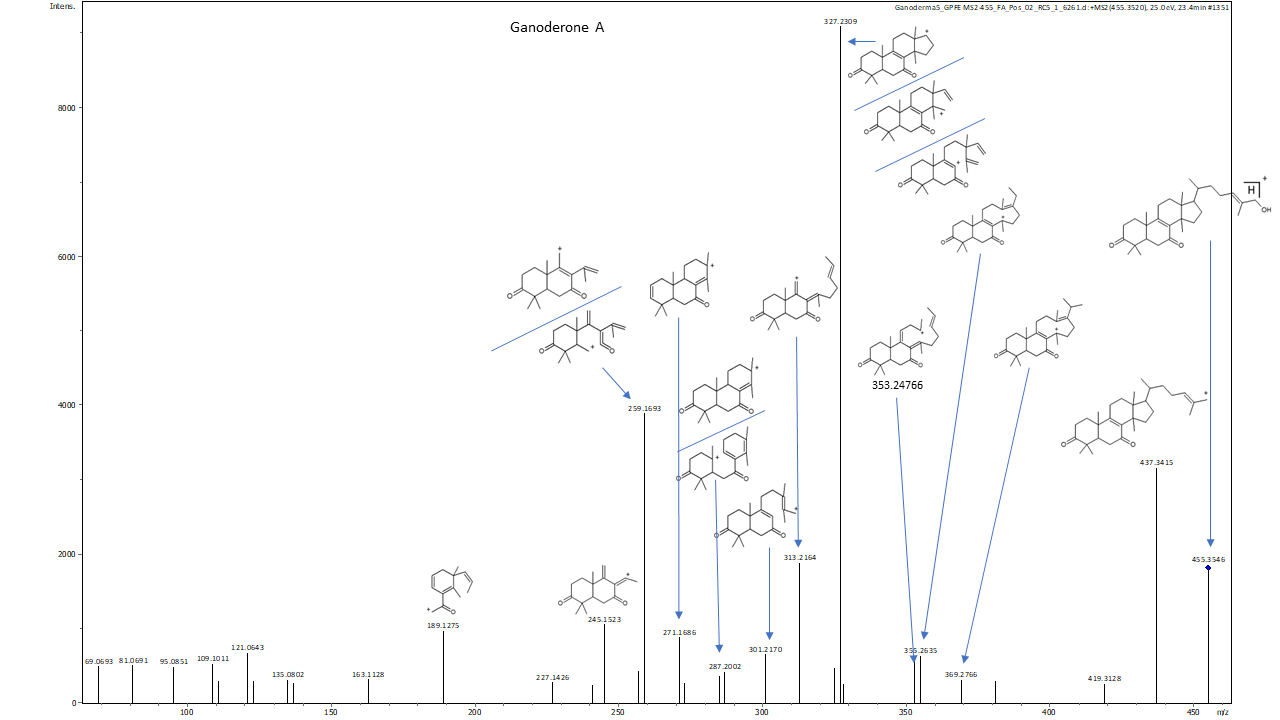


**Figure S9.** Annotated HRAM-MS^2^ spectrum of ganoderone A (m/z = 455.35 [M+H]^+^, RT = 23.41 min).


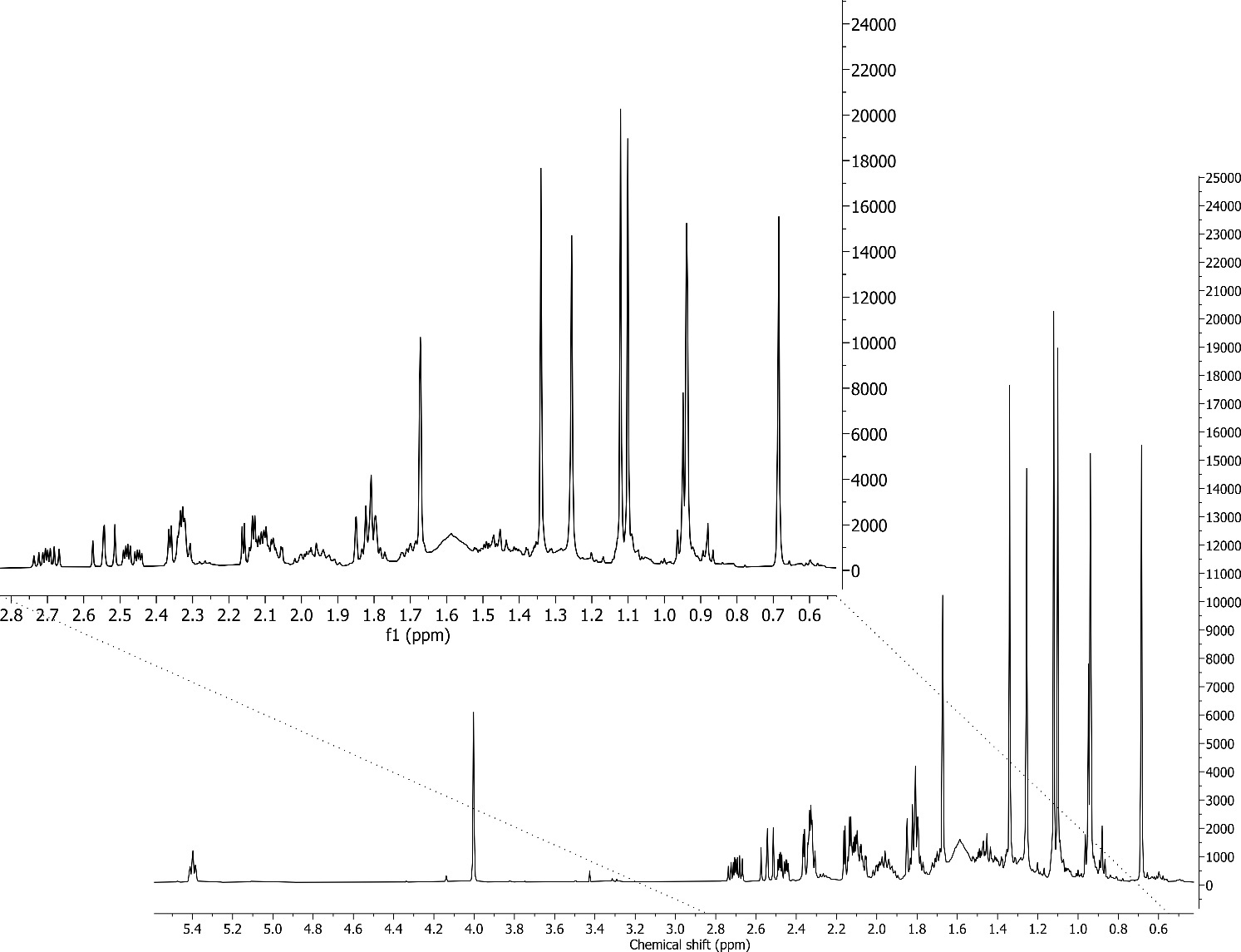


**Figure S10** ^1^H spectrum of purified ganoderone A in CDCl_3_ (500 MHz).


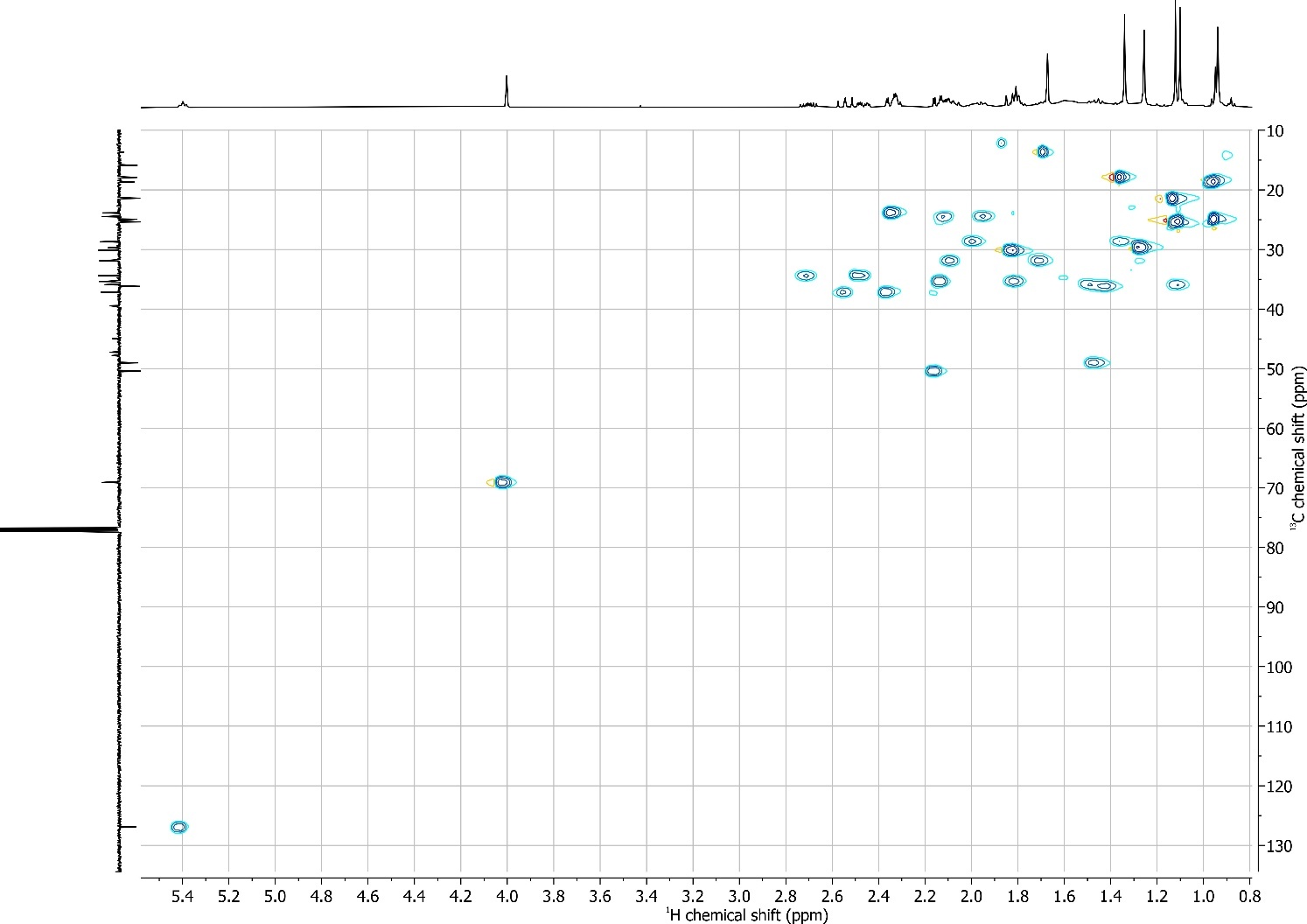


**Figure S11** HSQC spectrum of purified ganoderone A in CDCl_3_ (^1^H 500 MHz, ^13^C 125 MHz) .


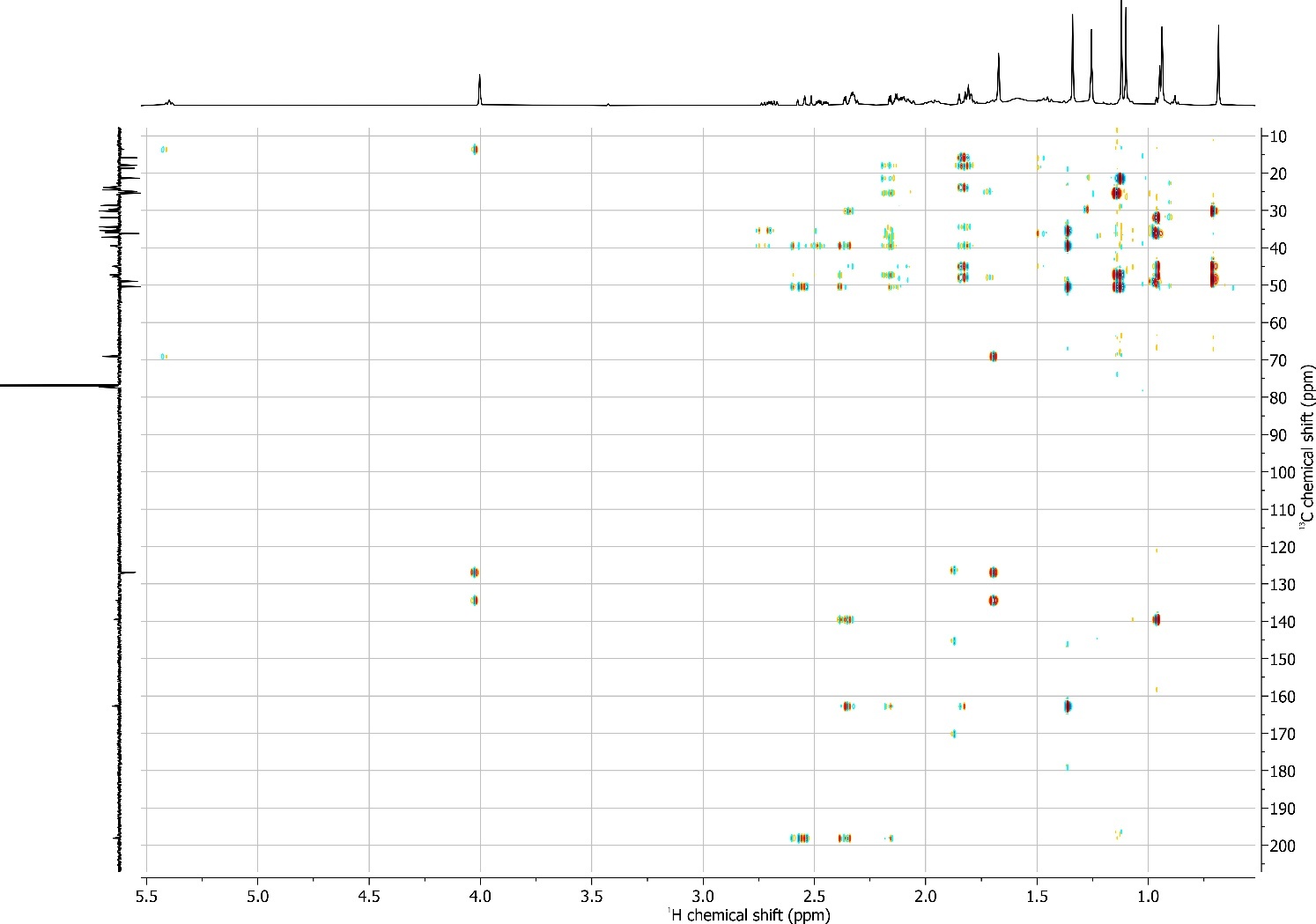


**Figure S12** HMBC spectrum of purified ganoderone A in CDCl_3_ (^1^H 500 MHz, ^13^C 125 MHz).


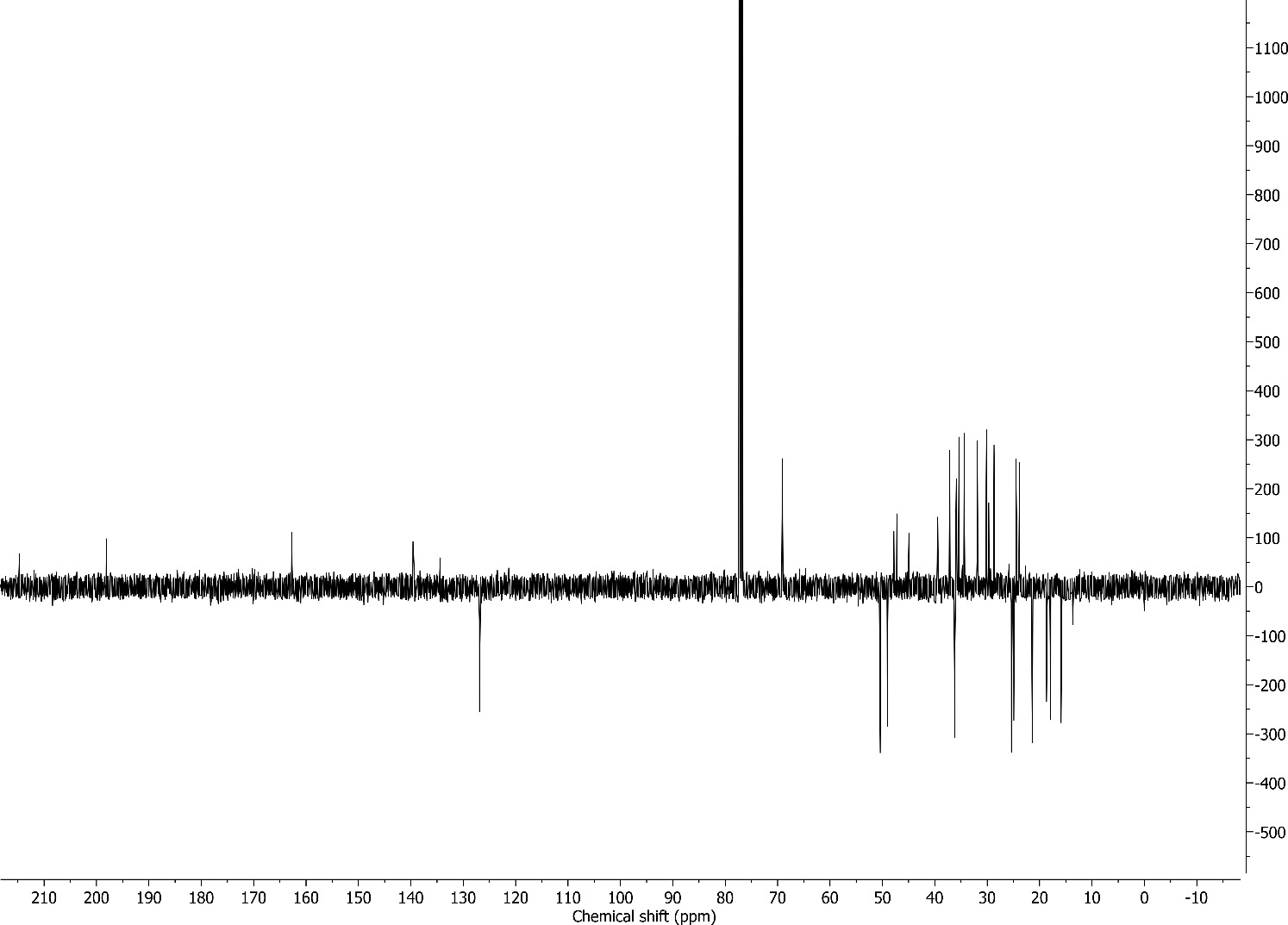


**Figure S13** ^13^C APT spectrum of purified ganoderone A in CDCl_3_ (125 MHz).


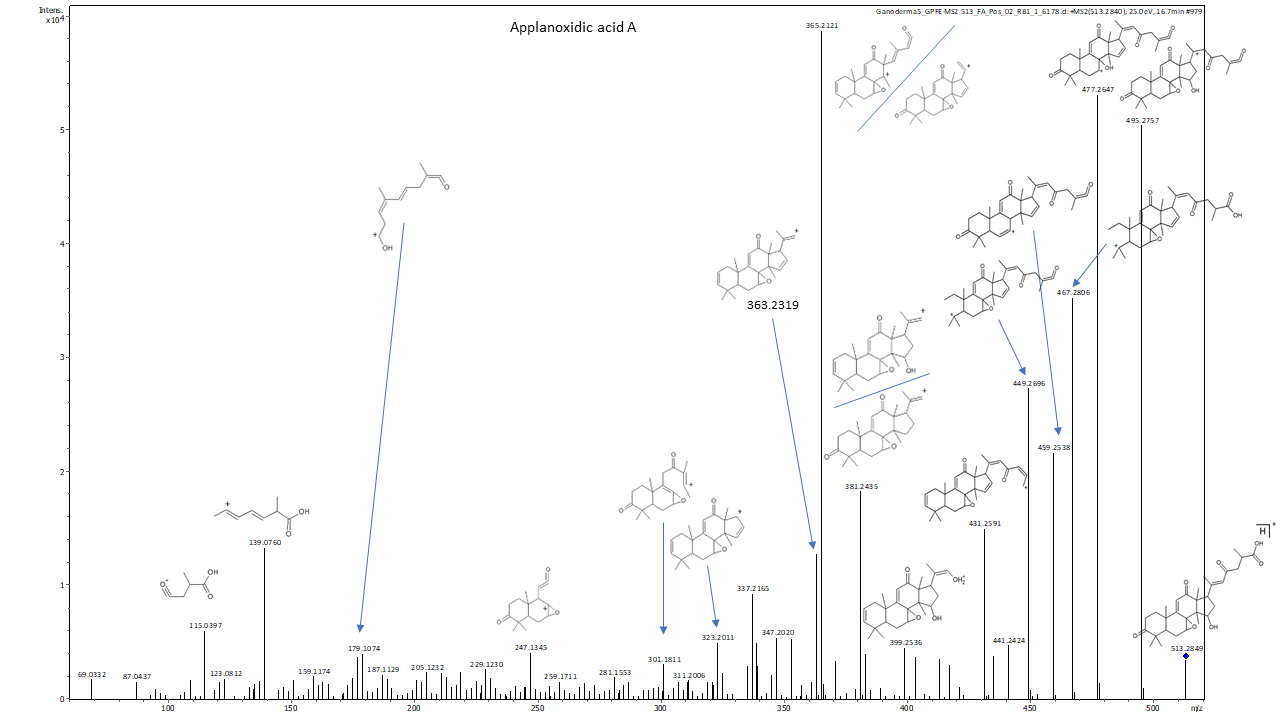


**Figure S14.** Annotated HRAM-MS^2^ spectrum of applanoxidic acid A (m/z = 513.29 [M+H]^+^, RT = 16.84 min).


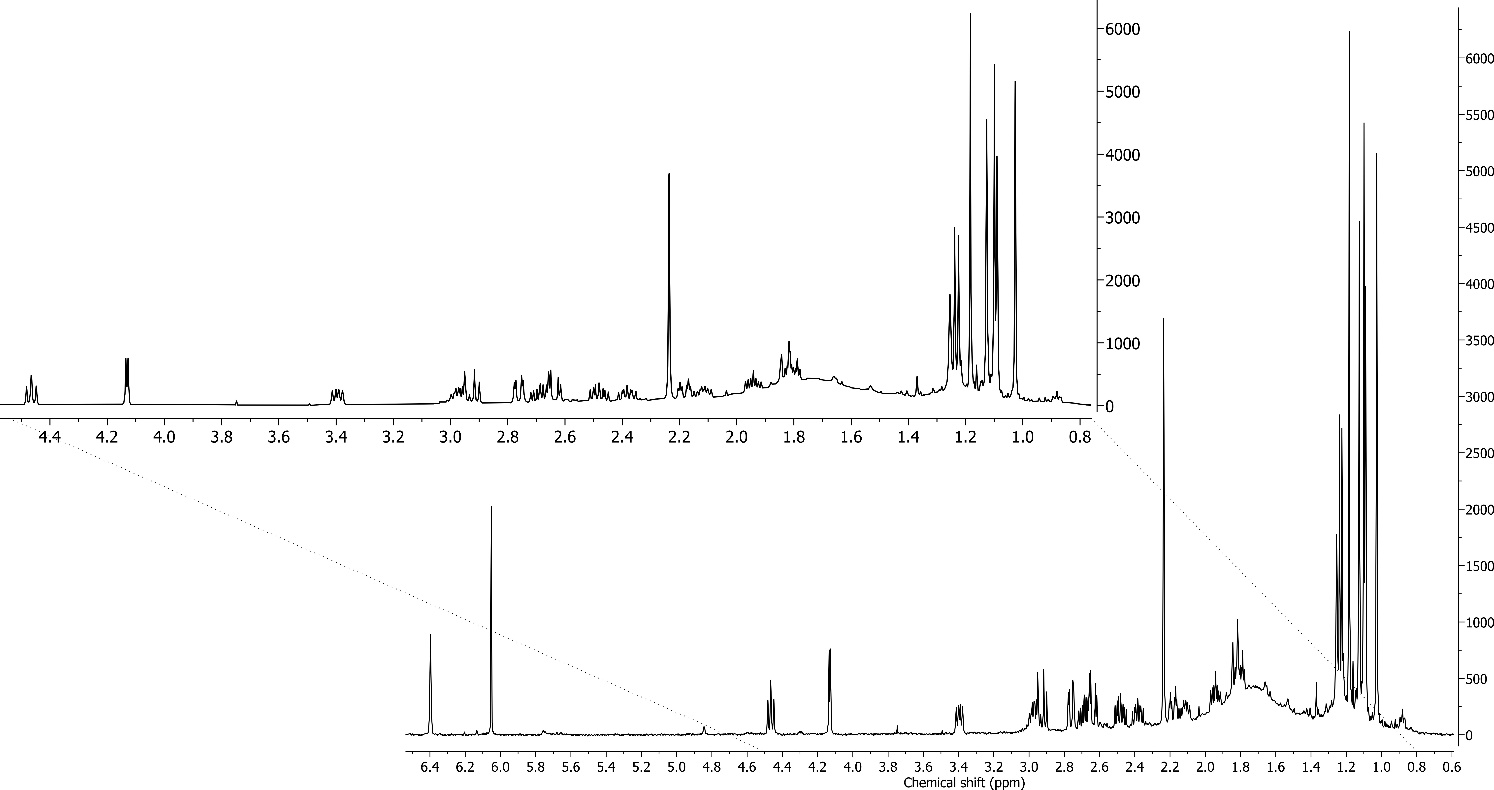


**Figure S15** ^1^H spectrum of purified applanoxidic acid A in CDCl_3_ (500 MHz).


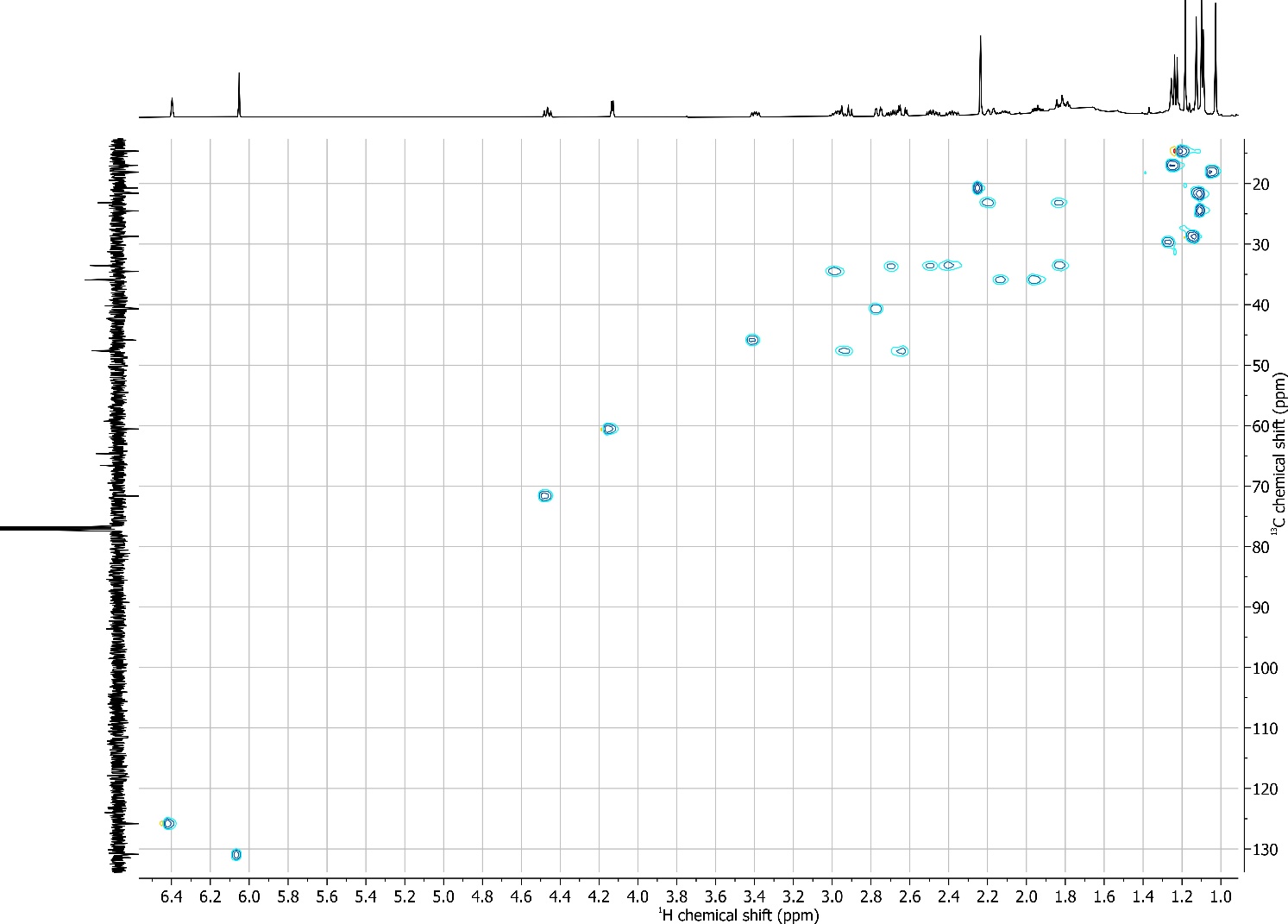


**Figure S16.** HSQC spectrum of purified applanoxidic acid A in CDCl_3_ (^1^H 500 MHz, ^13^C 125 MHz).


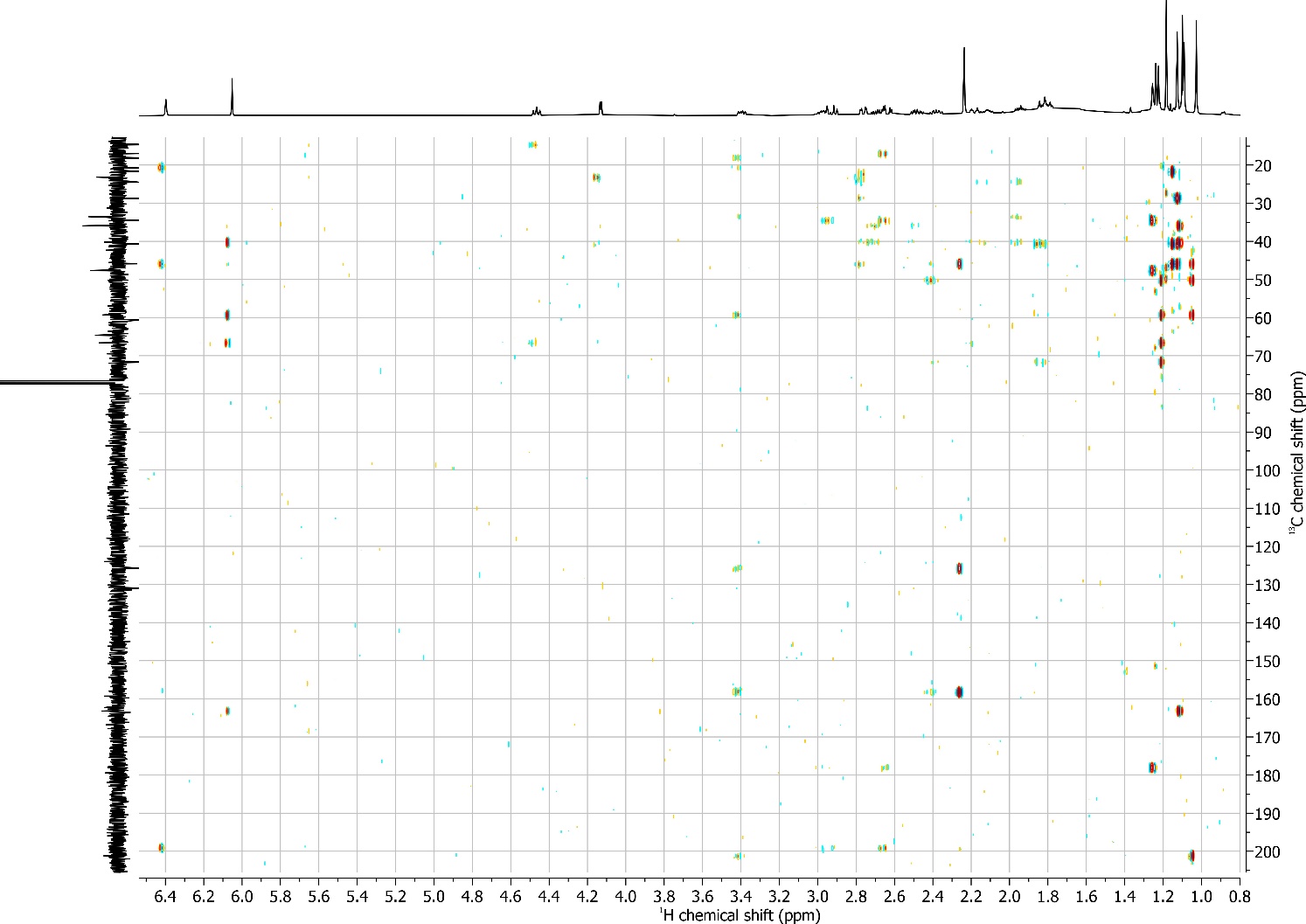


**Figure S17.** HMBC spectrum of purified applanoxidic acid A in CDCl_3_ (^1^H 500 MHz, ^13^C 125 MHz).


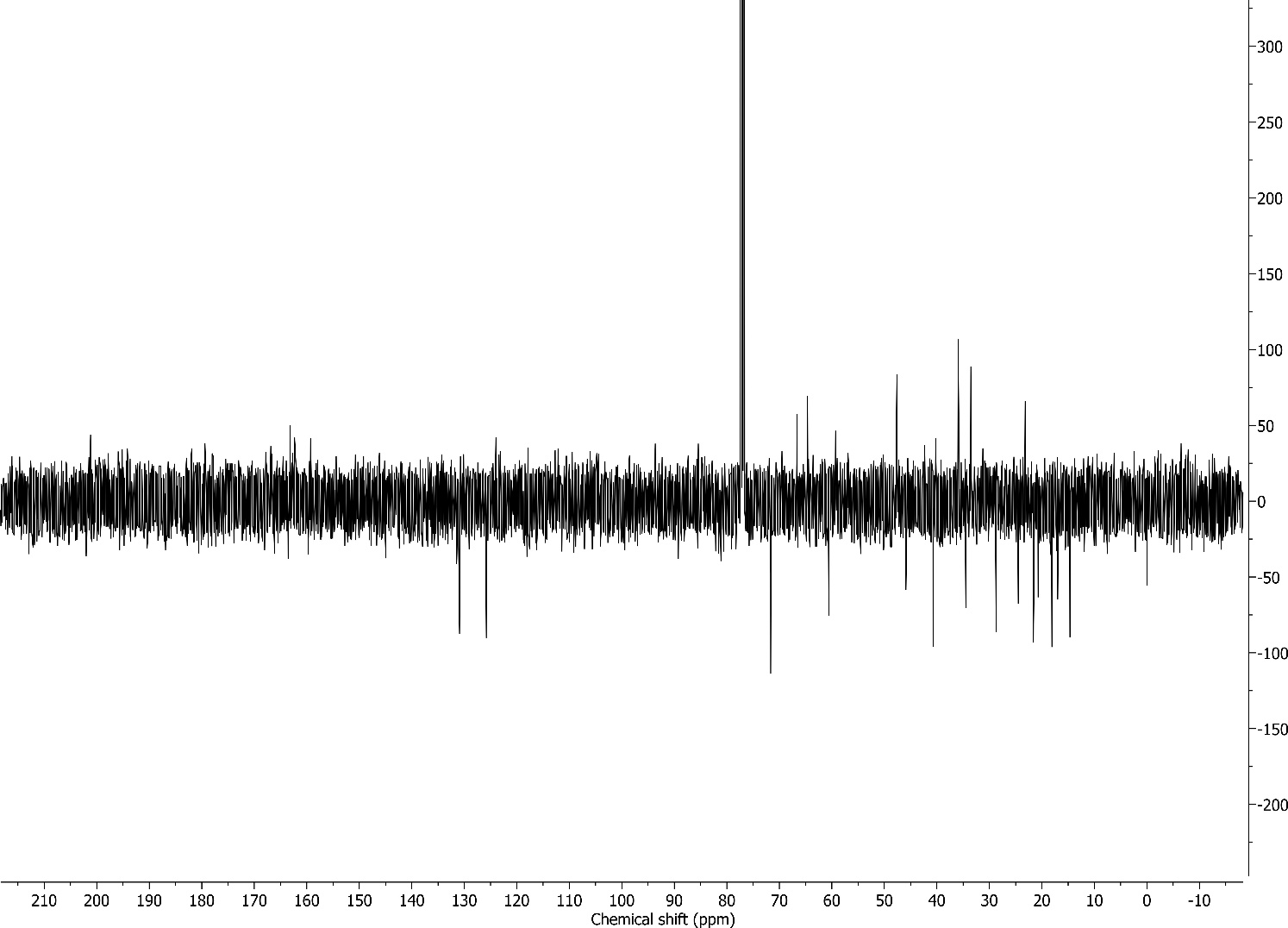


**Figure S18.** ^13^C APT spectrum of purified applanoxidic acid A in CDCl_3_ (125 MHz).


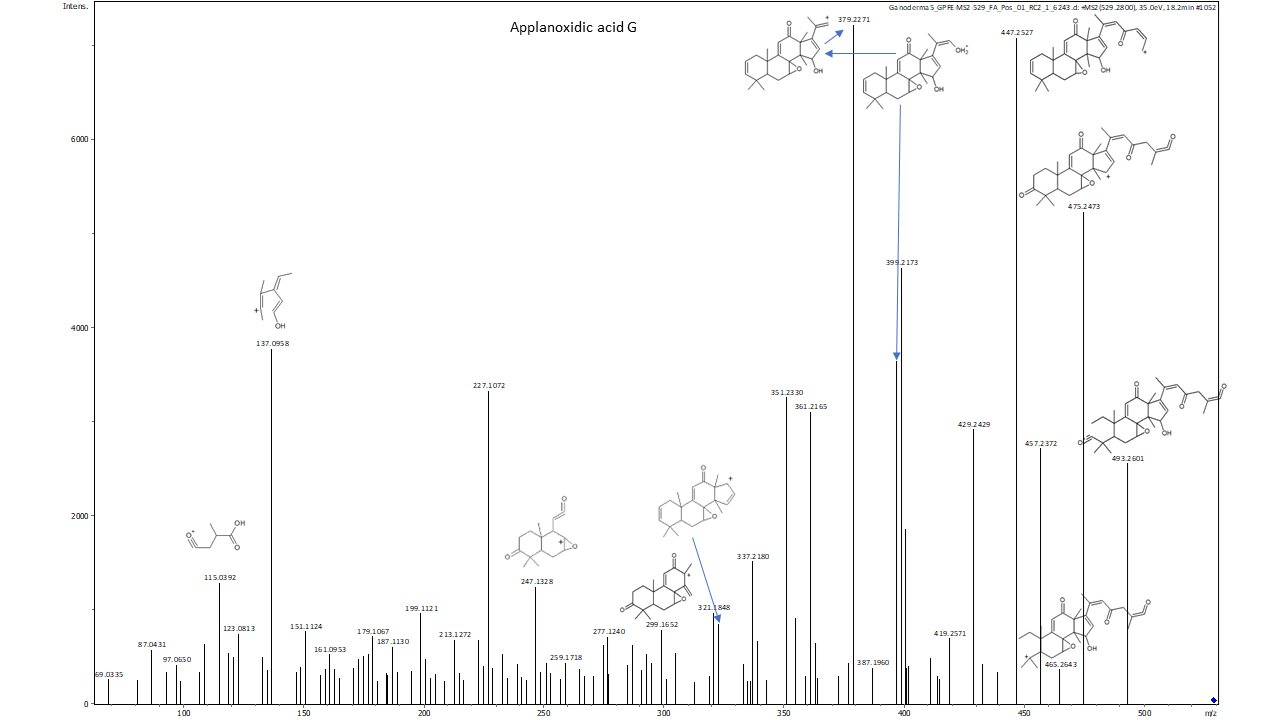


**Figure S19.** Annotated HRAM-MS^2^ spectrum of applanoxidic acid G (m/z = 529.28 [M+H]^+^, RT = 18.24 min).


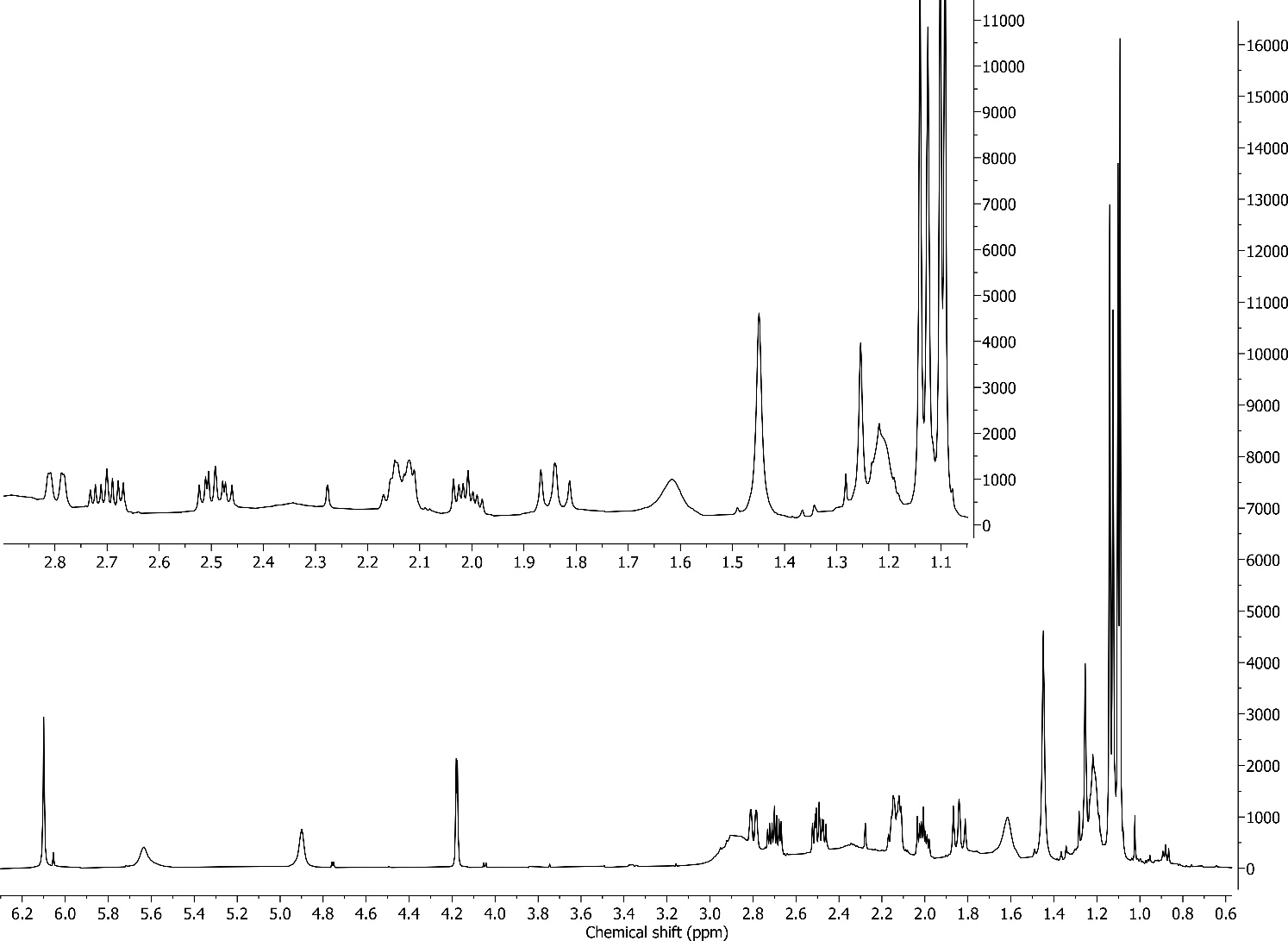


**Figure S20.** ^1^H spectrum of purified applanoxidic acid G in CDCl_3_ (500 MHz).


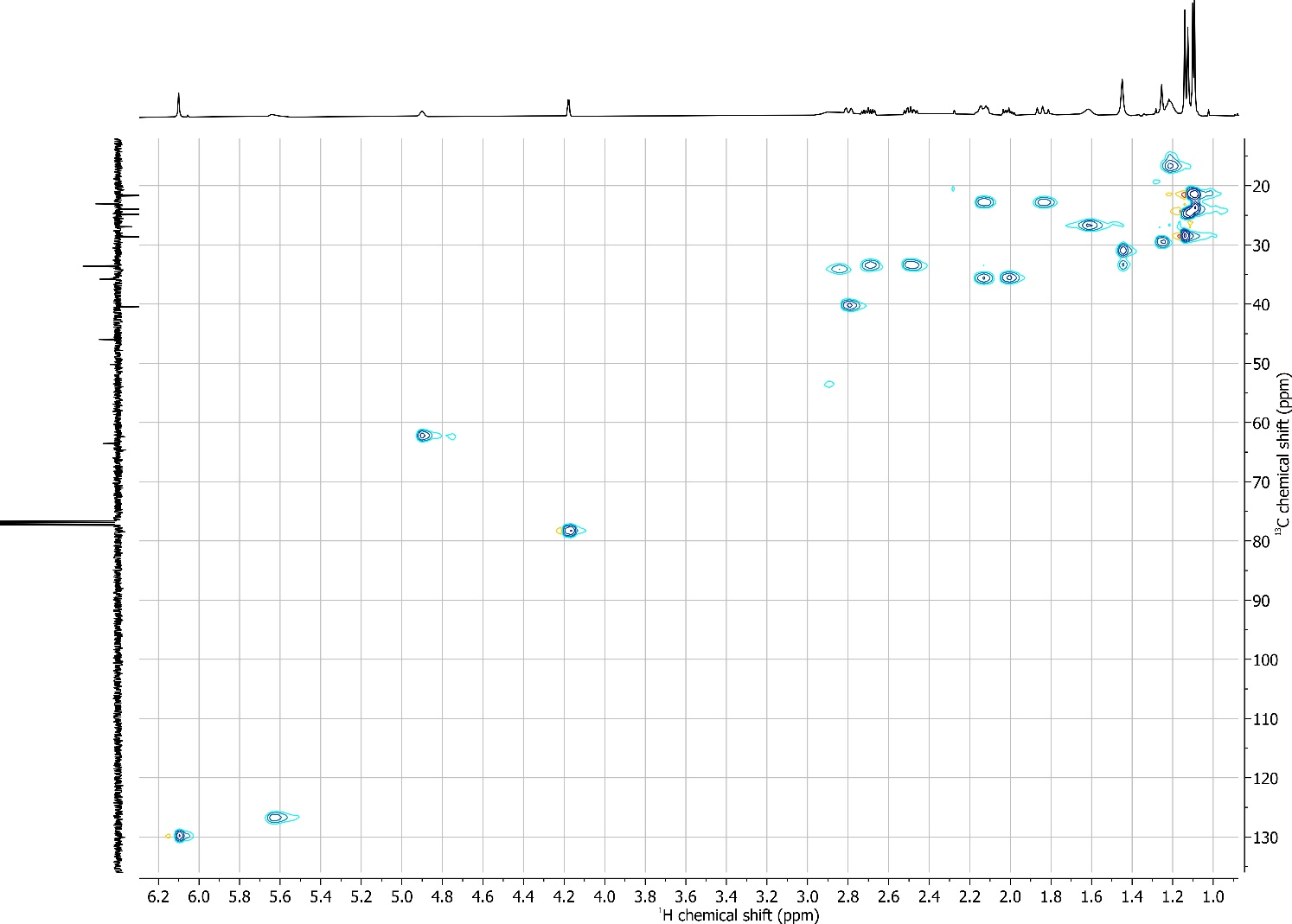


**Figure S21.** HSQC spectrum of purified applanoxidic acid G in CDCl_3_ (^1^H 500 MHz, ^13^C 125 MHz).


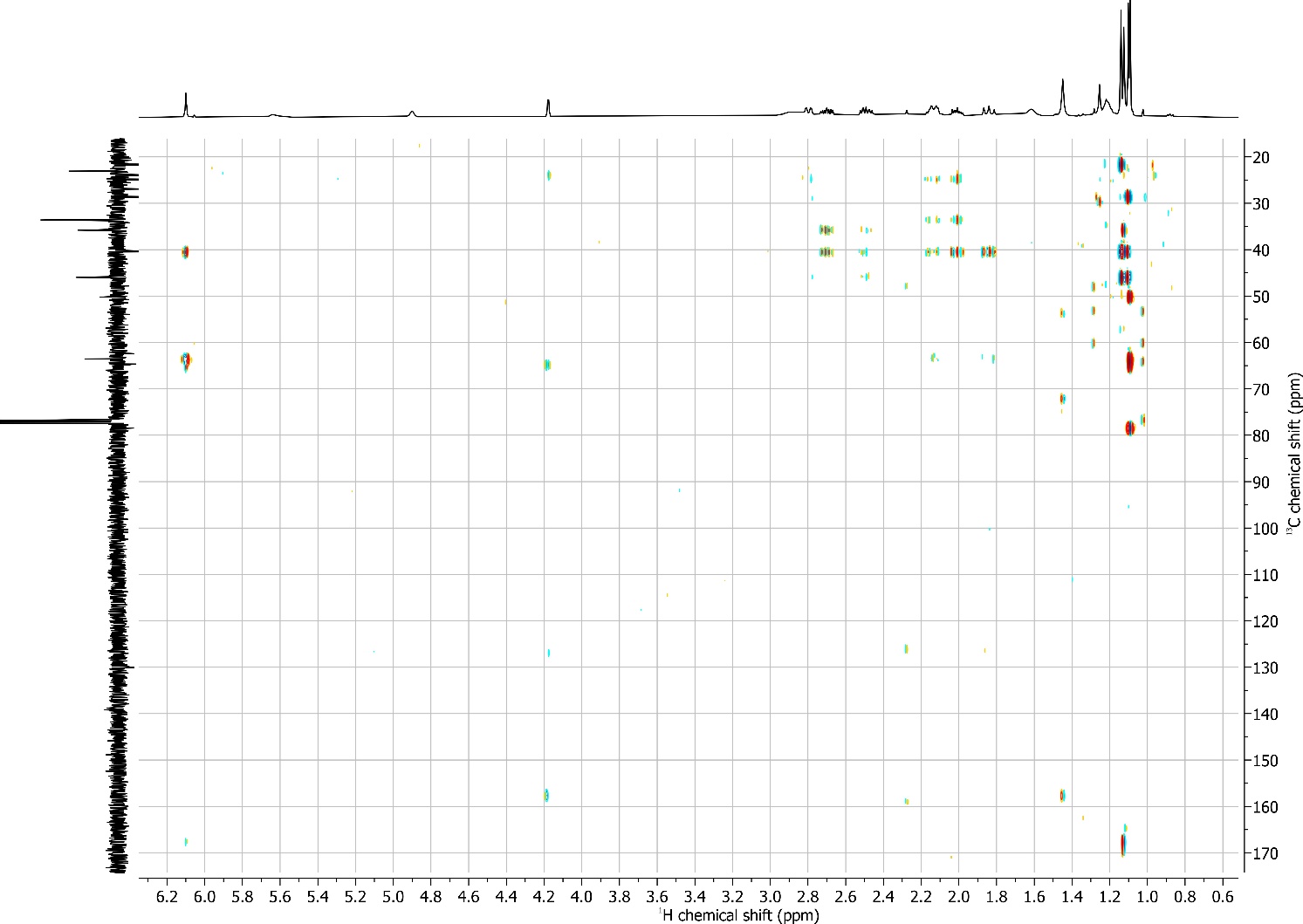


**Figure S22.** HMBC spectrum of purified applanoxidic acid G in CDCl_3_ (^1^H 500 MHz, ^13^C 125 MHz).


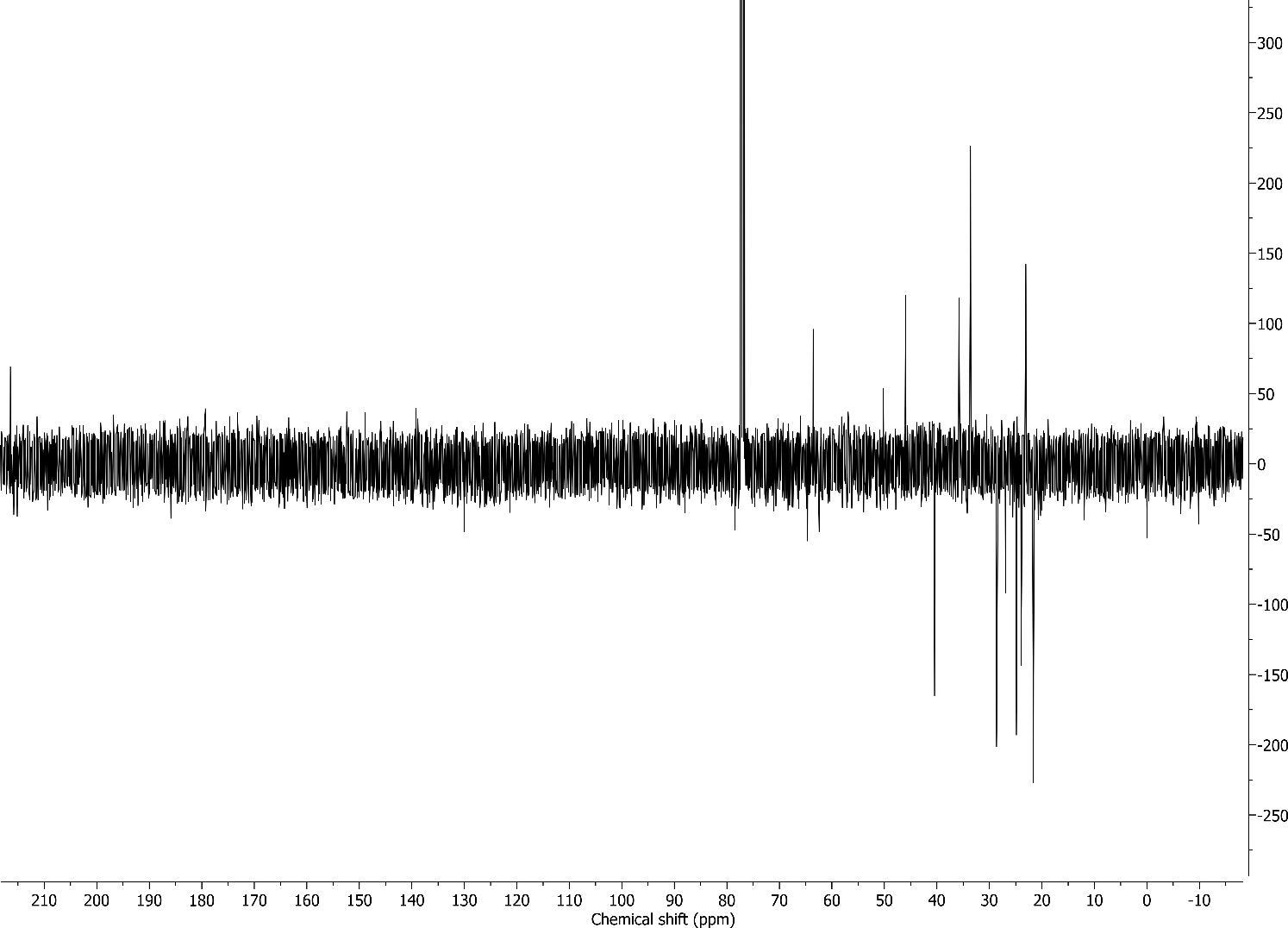


**Figure S23.** ^13^C APT spectrum of purified applanoxidic acid G in CDCl_3_ (125 MHz).

**Table S4** Content of monitored compounds in methanolic extracts of selected *Ganoderma* strains

|  | **Sample** | | |
| --- | --- | --- | --- |
| **Compound** | **GPFE** [μg/g d.w.] | **KZ-74** [μg/g d.w.] | **KZ-76** [μg/g d.w.] |
| applanoxidic acid A | 371.63 ± 10.01 | n.d. | n.d. |
| applanoxidic acid G | 1273.57 ± 61.56 | n.q. | n.d. |
| ganoderone A | 2065.77 ± 115.99 | n.q. | n.d. |
| ganoderone B | 945.61 ± 79.89 | n.q. | n.d. |

n.d. - not determined

n.q. - identified, but not quantified

**Table S5.** BET specific surface area, pore volumes, and pore sizes calculated from N_2_ adsorption-desorption isotherms for indicated materials.

| **Sample** | **SBET [m^2^/g]** | **Pore Volume [cm^3^/g]** | **Pore size [nm]** |
| --- | --- | --- | --- |
| MSN_cal_ | 1000.1 | 0.80 | 2.46 |
| MSN_GPFE_ | 108.1 | 0.09 | -- |

**Table S6.** Zeta potential and hydrodynamic potential of selected materials.

| **Nanomaterial** | **ζ potential (mV)** | **Hydrodynamic size (nm)** |
| --- | --- | --- |
| MSN_cal_ | −23.9 ± 0.6 | 169.4 ± 2.5 |
| MSN_GPFE_ | −10.6 ± 2.2 | 216.6 ± 8.7 |
